# *RYR1* Sequence Variants in Myopathies: Expression and Functional Studies in Two Families

**DOI:** 10.1155/2019/7638946

**Published:** 2019-04-21

**Authors:** Alberto Zullo, Giuseppa Perrotta, Rossana D'Angelo, Lucia Ruggiero, Elvira Gravino, Luigi Del Vecchio, Lucio Santoro, Francesco Salvatore, Antonella Carsana

**Affiliations:** ^1^CEINGE-Advanced Biotechnologies, Via Gaetano Salvatore 486, 80145 Naples, Italy; ^2^Department of Sciences and Technologies, University of Sannio, Via Port'Arsa 11, 82100 Benevento, Italy; ^3^Department of Neuroscience, Reproductive Sciences and Dentistry, University of Naples Federico II, Via Pansini 5, 80131 Naples, Italy; ^4^Department of Molecular Medicine and Medical Biotechnologies, University of Naples Federico II, Via Pansini 5, 80131 Naples, Italy

## Abstract

The skeletal muscle ryanodine receptor (RyR1), i.e., the Ca^2+^ channel of the sarco/endoplasmic reticulum (S/ER), and the voltage-dependent calcium channel Cav1.1 are the principal channels involved in excitation-contraction coupling in skeletal muscle.* RYR1* gene variants are linked to distinct skeletal muscle disorders, including malignant hyperthermia susceptibility and central core disease (CCD), mainly with autosomal dominant inheritance, and autosomal recessive myopathies with a broad phenotypic and histopathological spectrum. The age at onset of* RYR1*-related myopathies varies from infancy to adulthood. We report the identification of four* RYR1* variants in two Italian families: one with myopathy and variants c.4003C>T (p.R1335C) and c.7035C>A (p.S2345R), and another with CCD and variants c.9293G>T (p.S3098I) and c.14771_14772insTAGACAGGGTGTTGCTCTGTTGCCCTTCTT (p.F4924_V4925insRQGVALLPFF). We demonstrate that, in patient-specific lymphoblastoid cells, the c.4003C>T (p.R1335C) variant is not expressed and the in-frame 30-nucleotide insertion variant is expressed at a low level. Moreover, Ca^2+^ release in response to the RyR1 agonist 4-chloro-m-cresol and to thapsigargin showed that the c.7035C>A (p.S2345R) variant causes depletion of S/ER Ca^2+^ stores and that the compound heterozygosity for variant c.9293G>T (p.S3098I) and the 30-nucleotide insertion increases RyR1-dependent Ca^2+^ release without affecting ER Ca^2+^ stores. In conclusion, we detected and functionally characterized disease-causing variants of the RyR1 channel in patient-specific lymphoblastoid cells.

This paper is dedicated to the memory and contribution of Luigi Del Vecchio.

## 1. Introduction

The* RYR1* gene (OMIM *∗*180901) encodes the skeletal muscle ryanodine receptor (RyR1), which is the Ca^2+^ release channel of the sarcoplasmic reticulum (SR). RyR1 and the voltage-dependent L-type calcium channel Cav1.1 are the two principal channels involved in excitation-contraction coupling in skeletal muscle and are mechanically coupled. The activation of Cav1.1 by depolarization induces RyR1 to open and release calcium from the SR; this mechanism is called “depolarization-induced Ca^2+^ release.” RyR1 is also regulated by specific proteins, Ca^2+^, Mg^2+^, and ATP, and by pharmacological ligands.

Mutations in the* RYR1* gene are linked to several distinct skeletal muscle disorders, i.e., malignant hyperthermia susceptibility (MHS), central core disease (CCD), exertional rhabdomyolysis, King Denborough syndrome, late-onset axial myopathy, and congenital “core-rod myopathy,” all mainly with an autosomal dominant inheritance, whereas it is linked to subgroups of multiminicore disease (MMD), congenital fiber type disproportion, and centronuclear myopathy with an autosomal recessive inheritance [1]. The age at onset of* RYR1*-related myopathies varies from infancy to adulthood. In recent years, autosomal recessively inherited myopathies associated with sequence variants (SVs) in the* RYR1 *gene and with a broad phenotypic and histopathological spectrum have been reported [2–6]. Therefore, it has been suggested that* RYR1*-related myopathies are probably the most frequent form of congenital myopathies in several populations [3, 7, 8]. Malignant hyperthermia susceptibility is an autosomal dominant pharmacogenetic disorder that occurs in genetically predisposed subjects during general anesthesia and is induced by commonly used volatile anesthetics and/or the neuromuscular blocking agent succinylcholine. Triggering agents cause an abnormal increase in intracellular Ca^2+^ in skeletal muscle. A malignant hyperthermia (MH) attack, unless immediately recognized and treated with dantrolene, could be fatal. The gold standard for MH susceptibility (MHS) diagnosis is an in vitro contracture test (IVCT) on fresh muscle biopsies in which the differential contractile responses of MH-normal (MHN) and MHS muscles to caffeine and halothane are recorded [9, 10]. In about 70% of affected families, MHS is caused by mutations in the* RYR1* gene. Moreover,* RYR1* SVs, associated or possibly associated with MHS status, have been identified in patients who experienced exertional or stress-induced rhabdomyolysis, which are events similar to those occurring in MHS patients after exposure to anesthetics [11, 12]. Less than 1% of MHS cases are caused by mutations in the* CACNA1S* gene that encodes the *α*1S subunit of the voltage-dependent L-type calcium channel of the skeletal muscle, Cav1.1 [13–15].

Central core disease (MIM #117000) is a rare congenital myopathy characterized by muscle hypotonia and generalized weakness, delayed motor milestones, and skeletal malformations such as scoliosis and hip dislocation. However, marked clinical variability, often within the same family, has been recognized [16]. CCD is usually dominantly inherited, but some forms with more severe presentations have been reported to be associated with recessive inheritance [16]. Symptoms are usually present in infancy but may appear at any stage of life. The diagnosis of CCD is established by the histological analysis of muscle samples that usually reveals several central amorphous areas with sarcomere disorganization (“cores”) that lack mitochondria and oxidative enzyme activity in type 1 fibers. CCD patients are at risk of MHS; in fact, some patients experience clinical episodes of MH and/or were typed MHS by IVCT [17]. The identification and functional characterization of novel* RYR1* SVs are an aid to the diagnosis of MHS and CCD and to the elucidation of the molecular basis of the distinct pathophysiological characteristics of each* RYR1*-related disorder (drug-dependent hyperactivity in MHS, muscle weakness and core development in CCD, minicores in MMD, and histological anomalies in other* RYR1*-related myopathies). Thus far, more than 300 missense SVs have been identified in the* RYR1* gene (http://evs.gs.washington.edu/EVS/). Various methods have been developed to characterize the function of RyR1 variants [18–26]. The demonstration that the kinetic properties of the RyR1 channel are altered in MHS or in myopathic patients implicates the mutated channel in the pathogenesis of MHS and myopathies. Furthermore, genetic analysis can reveal cases of genotype/phenotype discordance, which are characterized by the presence of a causative mutation and an MHN-typed phenotype by IVCT [27, 28].

In this article, we report the identification of four* RYR1* SVs and the characterization of Ca^2+^ release in the lymphoblastoid cells of patients. We also report the results of expression studies in the same cell lines, demonstrating that some variant alleles are not expressed or are expressed at a low level, which may give some clues about the etiology of the disease.

## 2. Materials and Methods

### 2.1. Patients and IVCT Test

Informed consent for the sampling and the research use of biological material, for the genetic analysis, for the IVCT, for the treatment of personal information, and for the publication of this report was obtained from each patient according to the procedures established by the Declaration of Helsinki, the Italian law, and the Ethical Committee of the University of Naples “Federico II.”

### 2.2. Family NA-36

**Figure 1:**
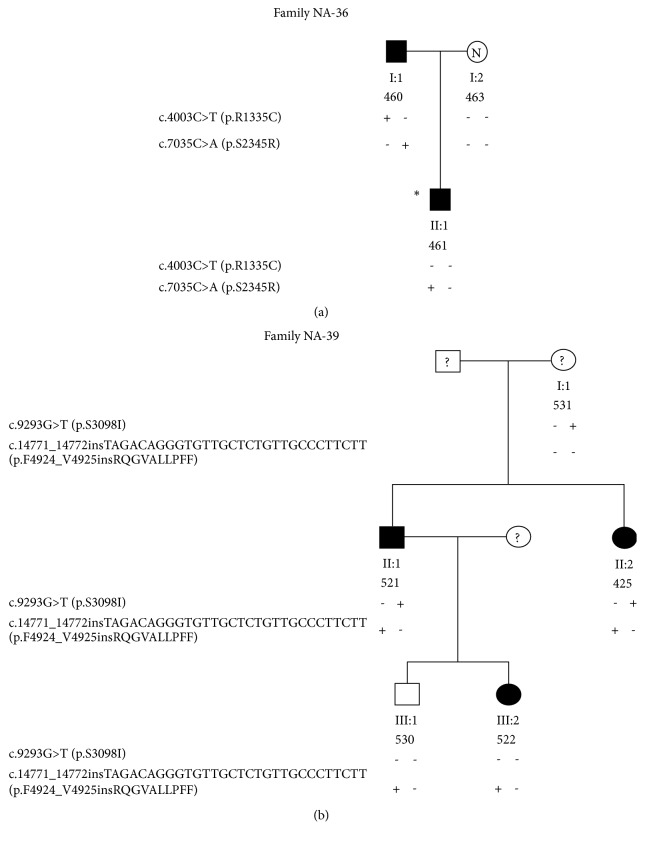
(a) Pedigree of the NA-36 family. Filled symbols indicate myopathy. The asterisk indicates the patient who experienced an MH episode; N indicates that the subject has been typed MHN by IVCT. (b) Pedigree of the NA-39 family. Filled symbols indicate CCD myopathy; the white symbol indicates a CCD-negative individual and the question mark indicates nontested individuals.

Patient 461 is a five-year-old boy who was referred to our Centre for an MH attack. At the age of two years, after anesthesia with sevoflurane for bilateral cryptorchidism, the creatine kinase (CK) level increased to 719 U/L (normal range 0-190 U/L) and the temperature to 40.2°C. Dantrolene was administered. At his last neurological evaluation, the patient exhibited dysmorphic facial features consisting of a prominent forehead, anteverted nares, arched palate, mandibular hypoplasia, palpebral ptosis, moderate widespread proximal muscle hypotrophy and hypotonia, and lumbar hyperlordosis. Myopathy was diagnosed. Patient 460 is the father of patient 461 (Figure 1(a)).

He exhibited short stature, mandibular prognathism, prominent eyebrows, sunken orbits, left testicular hypotrophy, weak and symmetrical osteotendinous reflexes, mild hypotrophy of the pelvic girdle and limb muscles, and, since childhood, kyphoscoliosis and thoracic lordosis. The electromyography (EMG) showed mild myopathic features. After the detection of high plasma levels of CK, ALT, and LDH, he underwent a biopsy of the biceps brachii muscle that revealed variability of fiber morphology with the presence of hypotrophic and atrophic (30%) fibers, a mild increase in connective tissue (10%) and rare ghost (5%) and degenerating (10%) fibers (Supplementary Figure S1). Overall, the histological findings were consistent with the diagnosis of primary myopathy. The IVCT failed because of the slow twitch of muscle fibers. Patient 461's mother (463, Figure 1(a)) does not show any sign of myopathy or facial dysmorphism and was typed MHN by IVCT.

### 2.3. Family NA-39

Patient 425 is a 46-year-old woman. She had delayed motor development with independent ambulation at five years, short stature, and severe dorsal scoliosis and lumbar lordosis since childhood. At the age of 17 years, she underwent a muscle biopsy and was diagnosed with CDD. At that age, restrictive respiratory insufficiency was observed, while the cardiologic assessment was normal. At her last neurological examination (2014), the patient had myopathic facies, mild weakness of the neck flexors, widespread muscle hypotrophy that was more severe in the proximal segments of limbs, waddling gait, equinus feet, lumbar hyperlordosis, and dorsolumbar right convex scoliosis; tricipital osteotendinous reflexes were absent. Ankle joints were blocked and heel walking was impossible. Gowers' maneuver was positive and the patient needed bilateral support to get up from a chair. Patient 521 is a 50-year-old man who has a clinical picture similar to that of his sister (patient 425), namely, myopathic facies, widespread muscle hypotrophy that was more severe in the proximal segments of limbs, waddling gait, lumbar hyperlordosis, dorsolumbar right convex scoliosis, and positive Gowers' maneuver. Patient 522, an 18-year-old woman, daughter of patient 521, had positive Gowers' maneuver, hyposthenia of the neck flexor, tricipitis and quadricipital muscles, mild hyposthenia of the shoulder girdle muscles, mild hyperlordosis, diminished tricipital osteotendinous reflexes, and absent patellar reflexes. A CCD diagnosis was advanced. Subject 530, the 21-year-old son of patient 521 at his last neurological examination (2016), was clinically asymptomatic. The clinical phenotype of the subject 531, the mother of patients 425 and 521 (Figure 1(b)), has not been ascertained.

### 2.4. IVCT

The standardized European IVCT was carried out as described elsewhere [29]. An increase in force of at least 0.2 g after exposure to either 2% halothane or 2 mM caffeine indicates MHS. Contracture thresholds at higher concentrations than these are considered normal.

### 2.5. Mutation Screening

Total RNA was extracted from peripheral blood lymphocytes using the QIAmp RNA Blood Mini kit (Qiagen, Hilden, Germany). Total RNA (2.5 *μ*g) was used to synthesize cDNA using the SuperScript VILO cDNA Synthesis kit (Invitrogen, Carlsbad, CA, USA). The* RYR1* cDNA was subsequently amplified in 49 overlapping fragments, using primers (Table S1) designed on the human* RYR1* transcript sequence (NM_000540.2), and sequenced with dye-terminator chemistry (Applied Biosystems) using an ABI3100 automated sequencer (Applied Biosystems, USA). Genomic DNA (gDNA) was extracted with the “Nucleon” procedure (Amersham, Little Chalfont, UK) from peripheral blood samples and immortalized lymphoblastoid cells. PCR primers for the amplification of the 106 exons of the* RYR1* gene and the intron boundary sequences were designed on the human* RYR1* genomic sequence (ENSG00000196218, NG_008866.1, accession numbers) as described elsewhere [25]. Nucleotide numbering was based on the reference cDNA sequence (NM_000540.2). Indirect evidence of the pathogenicity of the variants identified was based on the following conditions: the absence in 100 chromosomes from normal individuals of the same ethnic group checked by denaturing high-performance liquid chromatography (DHPLC) analysis as previously described [25]; the absence in the 1000 genomes resequencing project (https://www.ncbi.nlm.nih.gov/variation/tools/ 1000genomes); the prediction of their pathological character using three programs, namely, PMut (http://mmb.pcb.ub.es/PMut/), SIFT (http://sift.jcvi.org/), and PolyPhen-2 (http://genetics.bwh.harvard.edu/pph2/); the population allele frequencies determined using the ExAC Browser (Beta) database (http://exac.broadinstitute.org/) [30]; the potential pathogenic role of the variants identified using Alamut Focus version 0.9 (Interactive Biosoftware, Rouen, France). Alamut is a licensed software package available from Interactive Biosoftware (https://www.interactive-biosoftware.com). Secondary structure and hydropathy plot were predicted by the Emboss' Garnier software (http://www.bioinformatics.nl/cgi-bin/emboss/garnier/) and the Kyte & Doolittle plot (http://web.expasy.org/protscale/), respectively.

### 2.6. Mononuclear Cells and EBV-Transformed Cell Lines

Whole blood was collected in EDTA-treated tubes, and mononuclear cells were isolated by centrifugation on Ficoll-Hypaque (Sigma-Aldrich, Saint Louis, MO, USA) density gradient. For infection with Epstein-Barr virus (EBV), mononuclear cells were exposed to supernatants of the B95.8 cell line, according to standard procedures [31]. Cells were cultured in Iscove's Modified Dulbecco's Medium (Sigma-Aldrich, Saint Louis, MO, USA) supplemented with 20% fetal bovine serum (Hyclone Laboratories Inc., Logan, UT, USA) and 1% L-glutamine (Sigma-Aldrich, Saint Louis, MO, USA). The presence of* RYR1* SVs in the EBV-immortalized lymphoblastoid cells from patients was confirmed by DNA sequencing. Compared to cell lines carrying other* RYR1* SVs, the cell culture carrying the insertion variant prematurely displayed increased average cycle times and increased cell size, which are characteristic of replicative senescence in cultured cells [32]. At this stage, the cells were discarded.

### 2.7. Transcription Analysis

Total cellular RNA was extracted from EBV-immortalized B lymphocytes (1 x 10^7^ cells) using the QIAmp RNA Blood Mini kit (Qiagen, Hilden, Germany). The SuperScript III first-strand synthesis system (Invitrogen, Carlsbad, CA, USA) was used to synthesize first-strand cDNA from 2.5 *μ*g of total RNA. Real-time PCR reactions were carried out in 10 *μ*l reaction mixture containing cDNA (serial dilution 1:5, 1:10, 1:20, 1:40), 0.5 *μ*M of each primer, and 1x Platinum SYBR Green qPCR SuperMix (Invitrogen, Carlsbad, CA, USA). The* GAPDH* (glyceraldehyde-3-phosphate dehydrogenase, NM_001289745) was used as reference gene. Primers for* GAPDH* cDNA amplification were GAPDH- F: TGGTATCGTGGAAGGACTCAT and GAPDH- R: GAAGGCCATGCCAGTGAG (amplicon of 104 bp). Primers for* RYR1* cDNA amplification were* RYR1*-F: TCAACACGCCGTCTTTCCCT (c.14250-14269) and RYR1-R: GTTGGGCTTGCGCTCATTGT (c.14410-14391) (amplicon of 161 bp). The RYR1-F primer spans the 96-97 exon junction. The real-time PCR reactions were run in triplicate on a 7900 HT Fast Real-Time PCR System (Applied Biosystems, Foster City, CA, USA) using the following thermal profile: 50°C for 2 min, denaturation at 95°C for 10 min, 40 cycles with denaturation at 95°C for 15 s, annealing at 60°C for 30 s, and polymerization at 72°C for 30 s. A single fluorescence measurement was conducted at the end of the 72°C extension step. A melting curve program (94°C for 15 s, 60°C for 30 s, and 95°C for 15 s with continuous fluorescence measurements) was used to verify the uniformity of the PCR products. Derivative melting curves generated only a single peak, indicating that no nonspecific products or primer-dimers were present. Cycle threshold (Ct) values were determined using the software RQ manager 1.2 provided by the manufacturer (Applied Biosystems). The relative gene expression was calculated using the 2^-ΔΔCt^ method [33].

Semiquantitative PCR reactions were carried out to analyze the differential expression of the* RYR1* variant alleles in family NA-39 [34]. The following primers for* RYR1* and reference* ACTB* (*β* actin, NM_001101.3) cDNAs were used: RYR1- F: TCACATGTACGTGGGTGTCC (c.14658-14677) and RYR1-R: GCACTTGGTCTCCATATCCTCA (c.14877-14856, spanning the 103-104 exon junction); *β*-actin F: CGTCTTCCCCTCCATCGT and *β*-actin R: TGTTGGCGTACAGGTCTTTG. The F primers were labeled with fluorescent dyes PET and VIC, for the* RYR1* and the* ACTB* cDNAs, respectively. RT-PCR products (1 *μ*L) were separated on the ABI PRISM 3130 Genetic Analyzer (Applied Biosystems) at 15 kV, at 60°C for 25 min using the POP-7 polymer. Electrophoresis data were collected using the ABI PRISM 3130 Data Collection Software Application v1.1 (Applied Biosystems) and the areas of the electrophoresis peaks were calculated for each specific PCR product using ABI PRISM GeneScans Analysis Software v3.7.1 (Applied Biosystems).

### 2.8. Western Blot Analysis

The amount of the RyR1 protein in lymphoblastoid cells was determined by semiquantitative Western blot analysis using anti RyR1 rabbit polyclonal primary antibodies (kindly provided by Prof. V. Sorrentino, University of Siena, Italy) and anti *α*-actinin antibodies (Santa Cruz Biotechnology, Dallas, TX, USA) as previously described [35], and a secondary HRP-conjugated goat antibody (Bethyl Laboratories Inc., Montgomery, TX, USA). Specific antigen detection was performed using a chemiluminescent HRP substrate and quantified with a ChemiDoc XRS apparatus (Biorad, Hercules, CA, USA) and the Quantity 1 software (Biorad, Hercules, CA, USA).

### 2.9. Calcium Release Assay

The Ca^2+^ flux was measured by flow cytometry using the Fluo-3 and Fura Red Ca^2+^ sensitive fluorophores (Thermo Fisher Scientific, Waltham, MA, USA) according to Bailey and collaborators [36]. The fluorescence intensity of Fluo-3 increases upon binding Ca^2+^ and that of Fura Red decreases. Therefore, the Fluo-3/Fura Red fluorescence signal ratio is a ratiometric procedure for the analysis of intracellular Ca^2+^ fluxes. EBV-immortalized B lymphocytes were incubated with 2.6 *μ*M Fluo-3 AM and Fura-Red AM at 37°C and 5% CO_2_ for 30 min in Iscove's medium supplemented with 0.1% BSA (Sigma-Aldrich) and 1% L-glutamine. Subsequently, cells were resuspended in HBSS medium without Ca^2+^, 1 mM EGTA (Sigma-Aldrich) at 1x10^6^ cells/ml. A BD FACSAria cell sorter (BD, Franklin Lakes, NJ, USA) was used to measure Ca^2+^ fluxes. Intracellular dyes were excited at 488 nm and the emission signals were detected with the 530/30 nm (Fluo-3) and 610/20 nm (Fura Red) filters. Fluo-3, Fura-red, and Fluo-3/Fura-red emission signals were recorded for 2 min before stimulation to obtain baseline fluorescence values. Subsequently, 4-chloro-m-cresol (4-CmC) (Sigma-Aldrich) or thapsigargin (TG) (Sigma-Aldrich) were added to the cell suspension and the fluorescence signals were recorded for 5 min. A kinetic analysis of cell reactions was performed by splitting the Fluo-3 and Fura-red dot plot displays (fluorescence intensity vs time) into 50 gates. In each gate, the mean fluorescence values for Fluo-3 and Fura-red were determined using the Becton-Dickinson Analysis software. Microsoft Excel software (Microsoft Corporation, Redmond, WA, USA) was used to correct Fluo-3 and Fura-red signals for baseline values and to calculate Fluo-3/Fura Red ratios. The cellular responses to the drug (4-CmC or TG) were measured by integrating over time the Fluo-3/Fura Red ratio signals by SigmaPlot 11 software (SPSS Science, Chicago, IL, USA).

### 2.10. Statistical Analysis

Data were collected for each experimental condition from at least three independent experiments. To assess the statistical significance of intergroup differences (p<0.05), the standard two-tailed Student's t-test, or the one-way analysis of variance followed by Dunnett's test, was used. Results are presented as mean ± standard error of the mean (SEM). Statistical analysis and data plots were performed using SigmaPlot 11 software (SPSS Science, Chicago, IL, USA). Differences were considered significant when p<0.05.

## 3. Results

### 3.1. Identification and Characterization of RYR1 SVs

**Figure 2:**
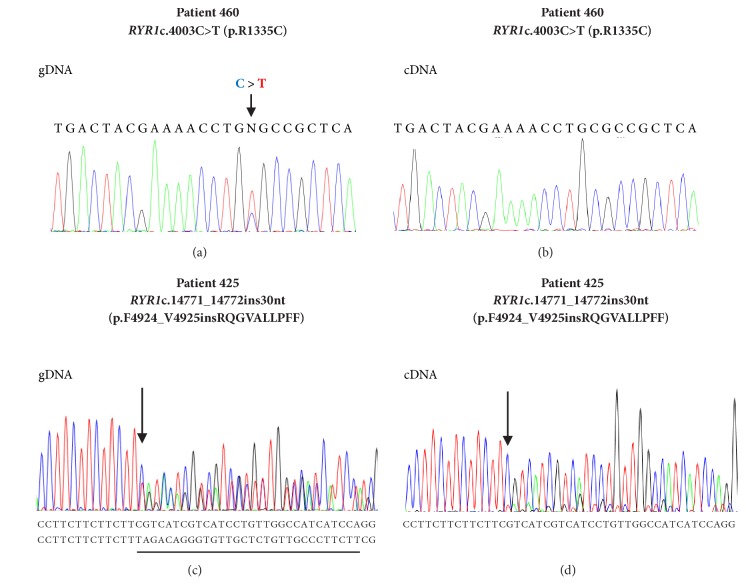
Sequence analysis of the variant* RYR1* c.4003C>T (p.R1335C) ((a) and (b)) in patient 460 and the variant* RYR1* c.14771_14772insTAGACAGGGTGTTGCTCTGTTGCCCTTCTT (p.F4924_V4925insRQGVALLPFF) ((c) and (d)) in patient 425 from gDNA ((a) and (c)) and cDNA ((b) and (d)) isolated from the patient's lymphocytes. The arrows in the nucleotide sequences indicate the position of the mutation; the sequence of the insertion in patient 425 is underlined.

The screening of the entire RYR1 cDNA and coding gDNA sequences revealed two RYR1 SVs at the heterozygous state in family NA-36 (Figure 1(a)). Substitutions c.4003C>T (exon 28, p.R1335C) and c.7035C>A (exon 44, p.S2345R) were identified in patient 460. Variant c.4003C>T (p.R1335C) was identified on gDNA but not on cDNA (Figures 2(a) and 2(b)). Accordingly, the c.7035C allele, which carries the c.4003T variant upstream, was barely detectable in cDNA of patient 460 (Supplementary Figure S2). No SNPs were detected on gDNA at cDNA primer annealing sites (cDNA amplification reaction 6, Supplementary Table S1), thereby excluding allele drop out in the cDNA PCR reaction.

Patient 461, who experienced an MH crisis after anesthesia with sevoflurane, inherited the c.7035C>A (p.S2345R) SV from his father (460) (Figure 1(a)).

The c.7035C>A (p.S2345R) SV was not found in 100 chromosomes from normal individuals of the same ethnic group by denaturing DHPLC analysis or in the 1000 genomes resequencing project (https://www.ncbi.nlm.nih.gov/variation/tools/ 1000genomes); this variant has been identified in a family with MHS and myopathy (hyperlordosis and toe walking) [5] and is predicted to be pathological by three prediction software programmes (PMut, http://mmb.pcb.ub.es/PMut/; SIFT, http://sift.jcvi.org/; PolyPhen-2, http://genetics.bwh.harvard.edu/pph2). Variant c.4003C>T (p.R1335C) was not found in 100 chromosomes from normal individuals of the same ethnic group by DHPLC analysis and is reported in the ExAC database (http://exac.broadinstitute.org/) with a minor allele frequency (MAF) of 4.1·10^−5^. Moreover, in silico analysis using the Alamut software package (https://www.interactive-biosoftware.com) showed that the c.4003C>T substitution alters the Exon Splicing Enhancer distributions and that allele c.4003T has a higher chance of exon 28 skipping than allele c.4003C (Supplementary Figure S3).

Similarly, two* RYR1* SVs at the heterozygous state were identified by screening the entire* RYR1* cDNA and coding gDNA sequences of patient 425 who is the CCD proband of family NA-39 (Figure 1(b)), namely, the c.9293G>T (exon 63, p.S3098I) substitution and the insertion variant c.14771_14772insTAGACAGGGTGTTGCTCTGTTGCCCTTCTT (exon 102, p.F4924_V4925insRQGVALLPFF). In patient 425, sequence analysis showed a disequilibrium between the cDNA levels of the wild-type and of the variant sequences (Figure 2(d)). In fact, both the cDNA and the gDNA electropherograms showed two overlapping sequences, but, in the cDNA, the peaks of the mutated sequence were much lower than those of the wild type. On the contrary, in the gDNA, the heights of the peaks of the mutated sequence were similar to those of the wild type (Figure 2(c)). Two ARMS reactions with two insertion-specific primer pairs designed to amplify the allele with insertion confirmed the presence of the insertion variant (Supplementary Figure S4).

Similar electrophoretic patterns were obtained with cDNA and gDNA from patient 521, who has the same* RYR1* haplotype as his sister (patient 425), and his children (patients 522 and 530) (data not shown), who inherited only the insertion variant from the father (Figure 1). These data indicate low levels of the c.14771_14772insTAGACAGGGTGTTGCTCTGTTGCCCTTCTT (p.F4924_V4925insRQGVALLPFF) mutated mRNA molecules. Interestingly, we found that the first 25 nt of the 30 nt inserted sequence corresponds to an inverted and complementary sequence located at* RYR1* intron 105 (158553-158529, NG_008866.1, Supplementary Figure S5). Sequencing of the entire* RYR1* genomic region spanning from intron 102 to exon 106 of CCD patient 425 (data not shown) did not reveal a deletion of the transposed sequence or any additional SVs in the mutated DNA that could have generated rearrangement breakpoints. Therefore, we hypothesized that this insertion variant could have arisen from a replicative transposition involving the intron sequence replication and its inversion and transfer to the new site. The 10-amino acid insertion variant c.14771_14772insTAGACAGGGTGTTGCTCTGTTGCCCTTCTT (F4924_V4925insRQGVALLPFF) lies in the S6 transmembrane segment (amino acids 4914-4937) of the RyR1 structure model proposed by Zalk and collaborators [37]. Secondary structure prediction using the Emboss Garnier software (http://www.bioinformatics.nl/cgi-bin/emboss/garnier/) and the analysis of hydropathy using the Kyte & Doolittle method (http://web.expasy.org/protscale/) showed that the 10-amino acid insertion causes an interruption in an *α*-helix structure (Supplementary Figure S6) and modifies the hydropathy plot of the S6 segment (Supplementary Figure S7). The c.9293G>T (p.S3098I) variant identified in family NA-39 was not detected in 100 alleles of normal individuals of the same ethnic group by DHPLC analysis or identified as a polymorphism in the 1000 genomes sequencing project (https://www.ncbi.nlm.nih.gov/variation/tools/ 1000genomes) but is reported in the ExAC database (http://exac.broadinstitute.org/) with a MAF of 8.2·10^−6^ and probably affects the function of the RyR1 channel. In fact, the c.9293G>T (p.S3098I) substitution was predicted to be pathological by three software programmes (PMut, http://mmb.pcb.ub.es/PMut/; SIFT http://sift.jcvi.org/; PolyPhen-2 http://genetics.bwh.harvard.edu/pph2).

### 3.2. Transcription Analysis

**Figure 3:**
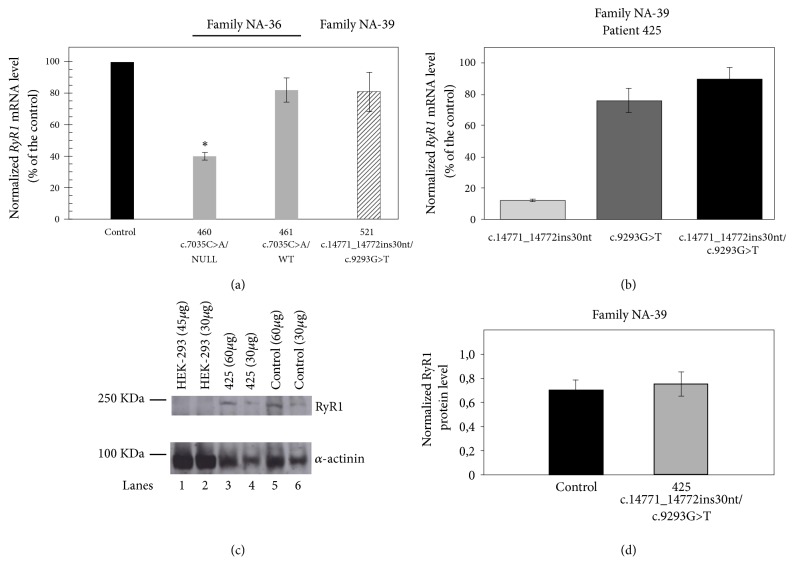
Expression of* RYR1* variant alleles. (a) Relative quantification of* RYR1* mRNA isolated from immortalized B-lymphocytes of patients 460, 461 (Family NA-36), and 521 (Family NA-39. Real-time PCR was performed on cDNAs of patients and of a control.* GAPDH *was used as reference gene. The relative expression was calculated using the 2^-ΔΔCt^ method. Data are presented as mean ± standard error of the mean (SEM). Statistical analysis was performed by one-way analysis of variance followed by Dunnett's test (*∗*p< 0.05). (b) To analyze the relative expression of the two variant alleles identified in family NA-39, the PCR products from cDNAs of patient 425 were analyzed by capillary gel electrophoresis, as described in Materials and Methods.* RYR1 *mRNA levels in the CCD patient 425 (normalized by *β*-actin mRNA levels) are expressed as percentages of the control. Each experiment was performed in triplicate. Data are presented as mean ± SEM. (c) and (d) RyR1 protein levels of patient 425 determined by semiquantitative Western blot. (c) Representative Western blot. Homogenates from HEK293 cells (lanes 1 and 2) as a control not expressing RyR1, from lymphoblastoid cells of patient 425 (lanes 3 and 4), and from the control (lanes 5 and 6) were loaded on an SDS-polyacrylamide gel and processed as described in Materials and Methods. (d) The RyR1 protein levels in the control and in patient 425 were normalized by *α*-actinin levels. Data are presented as mean of three experiments. Error bars show the SEM. Statistical analysis was performed by the two-tailed Student's t-test (p=0.617).

The* RYR1* gene is expressed in human B-lymphocytes [38] and Ca^2+^ homeostasis is altered in B cells of MHS and CCD patients. Therefore, this cell system can be used as a model to test the effect of* RYR1* SVs when muscle samples are not available. We, therefore, analyzed the expression of the* RYR1* variants identified in retrotranscribed RNA samples isolated from patients' immortalized B lymphocytes. In family NA-36, real-time PCR analysis showed that the total* RYR1* mRNA levels in patient 460, carrying variants c.4003C>T (p.R1335C) and c.7035C>A (p.S2345R), and in patient 461, carrying variant c.7035C>A (p.S2345R), were about 40% (n=11, p-value < 0.001) and 82% (n=11, p-value > 0.05), respectively, of the levels of the control sample (Figure 3(a)). In family NA-39, the total* RYR1* mRNA level of patient 521, carrying variants c.9293G>T (p.S3098I) and c.14771_14772insTAGACAGGGTGTTGCTCTGTTGCCCTTCTT (p.F4924_V4925insRQGVALLPFF), did not differ significantly from the levels of the control sample (81%, n=8, p-value > 0.05) (Figure 3(a)). As shown in Figure 2(d), the electrophoretic patterns of cDNA sequencing performed on patients of family NA-39 indicated low expression of the 30 nt insertion allele (Figure 2(d) and data not shown). Therefore, we measured* RYR1* mRNA levels of the 30 nt insertion variant by a semiquantitative PCR analysis [34] on cDNA of patient 425 (family NA-39). To this aim, we amplified the* RYR1* fragment containing the insertion site (c.14658-14877). The amplicons from cDNA, separated by capillary gel electrophoresis, revealed two peaks, one of 220 bp and the other of 250 bp, which correspond to the expected lengths of the* RYR1* alleles without and with the 30 nt insertion, respectively. The total* RYR1* mRNA levels of CCD patient 425 were about 89% of those of the control (n=3). In particular, the* RYR1* mRNA alleles with and without the 30 nt insertion represented about 13% and 76%, respectively, of the total* RYR1* mRNA levels (Figure 3(b)). These data demonstrate that the levels of mRNA with the insertion variant are low in CCD patient 425, but this unbalanced expression does not basically affect the total* RYR1* expression levels. Accordingly, the* RyR1* protein content determined by semiquantitative Western blots (Figures 3(c) and 3(d)) in lymphoblastoid cells from patient 425 was not statistically different from that of the control (n = 3, p-value > 0.05).

### 3.3. Calcium Release Measurements

**Figure 4:**
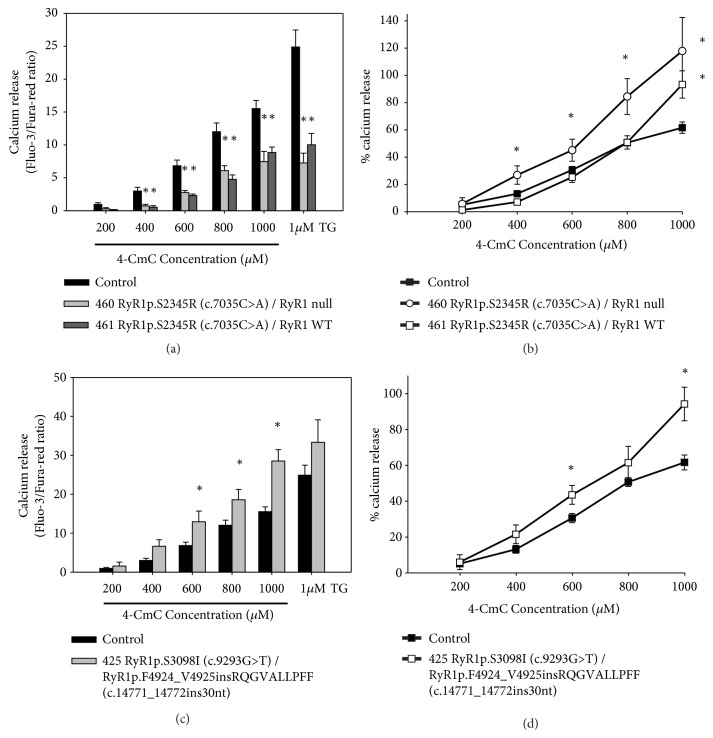
4-CmC-induced Ca^2+^ release in lymphoblastoid cells from patients 460, 461 ((a) and (b)), and 425 (c) and (d)) expressing the* RYR1* c.7035C>A (p.S2345R ), the* RYR1 *wt/*RYR1* c.7035C>A (p.S2345R), and the* RYR1* c.9293G>T (p.S3098I)/*RYR1* c.14771_14772insTAGACAGGGTGTTGCTCTGTTGCCCTTCTT (p.F4924_V4925insRQGVALLPFF) variant alleles, respectively, and two control cell lines. Cells were stimulated with 4-CmC (200 *μ*M to 1000 *μ*M) or 1 *μ*M TG. Ca^2+^ release was measured at each concentration by calculating the integral of the Fluo-3/Fura Red ratio signal over time ((a) and (c)) and expressed as a percentage of the TG-triggered Ca^2+^ release ((b) and (d)). Experiments were carried out at least three times. Data are reported as mean ± standard error of the mean (SEM). Statistical analysis of responses of patient cell lines with respect to the average of the two control cell lines was performed by the two-tailed Student's t-test or by one-way analysis of variance followed by Dunnett's test (*∗*p< 0.05).

We investigated Ca^2+^ release in response to stimulation with 4-chloro-m-cresol (4-CmC) or thapsigargin (TG) in immortalized B-lymphocytes from patients 460, 461 (family NA-36), and 425 (family NA-39) and from two unrelated control cell lines using a flow cytometry technique. No statistically significant difference was assessed between the calcium release measurements from the two control cell lines. Therefore, data from the two control cell lines have been averaged and presented in the plots as a single control group. 4-CmC specifically activates the RyR1 channel [39, 40]. TG is a pump blocker of the sarco/endoplasmic reticulum calcium ATPase (SERCA) that hinders Ca^2+^ uptake into the sarco/endoplasmic reticulum (SR/ER), thereby allowing passive leakage of Ca^2+^ from the SR/ER and Ca^2+^ store [41, 42]. As shown in Figure 4(a), 4-CmC-triggered and TG-induced Ca^2+^ releases were significantly lower in immortalized B-lymphocytes from patients 460 and 461 (family NA-36) than in control cells. Moreover, resting intracellular calcium concentration in immortalized B-lymphocyte from patients 460 and 461 was not different from controls (Supplementary Figure S8; p >0.05). Patient 460 expressed only the* RYR1* c.7035C>A (p.S2345R) allele, whereas patient 461 expressed the* RYR1* wt and the* RYR1* c.7035C>A (p.S2345R) alleles. Given the finding that the signal obtained with the SERCA blocker reflects the size of the rapidly releasable intracellular Ca^2+^ stores [43], our results indicate depletion of the Ca^2+^ store. However, Ca^2+^ release triggered by 4-CmC, expressed as a percentage of the TG-induced Ca^2+^ release [44], was significantly higher in B lymphoblastoid cells from patient 460 (from 400 to 1000 *μ*M 4-CmC) and from patient 461 (at 1000 *μ*M 4-CmC) versus the controls (Figure 4(b)). The higher sensitivity to 4-CmC of cell lines from patient 460 versus the control also emerges from the shift to the left of the dose-response curve (Figure 4(b)).

Ca^2+^ release triggered by 600-1000 *μ*M 4-CmC was significantly higher in immortalized B-lymphocytes from patient 425 (family NA-39), who expressed the* RYR1* c.9293G>T (p.S3098I) and the* RYR1* c.14771_14772insTAGACAGGGTGTTGCTCTGTTGCCCTTCTT (p.F4924_V4925insRQGVALLPFF) variant alleles, than in the control cells, whereas the TG-induced Ca^2+^ release did not differ significantly (Figure 4(c)). Moreover, the dose/response curve of the cells from patient 425 showed a shift to the left compared to the curve from the controls (Figure 4(d)). Resting intracellular calcium concentration in immortalized B-lymphocyte from patients 425 was not different from controls (Supplementary Figure S8; p>0.05).

## 4. Discussion

Here we report two Italian families, one with myopathy and another with CCD, associated with* RYR1* variants and* RYR1* unbalanced expression in lymphoblastoid cells. The expression of the c.4003C>T variant (family NA-36), which would correspond to the p.R1335C substitution, was abolished (Figures 2(b) and 3(a), Supplementary Figure S2). The c.4003T variant is predicted to have a higher chance of exon 28 skipping. Alternative splice variant lacking exon 28 would harbor a premature stop codon, thus possibly leading to a nonsense-mediated mRNA decay [45].

The expression of the c.14771_14772insTAGACAGGGTGTTGCTCTGTTGCCCTTCTT (p.F4924_V4925insRQGVALLPFF) (family NA-39) variant was reduced to about 13% of that of the control (Figure 3(b)).* RYR1* missense alleles with null or reduced expression, mainly analyzed in muscle samples, have been reported in patients with* RYR1*-related myopathies [4, 46–52]. Epigenetic changes regulating* RYR1* expression have been described in muscle but not in cultured myotubes [49]. We found that the expression of missense alleles was reduced in lymphoblastoid cells derived from our patients; however, our* RYR1* expression results should be verified in skeletal muscle cells. Unfortunately, muscle samples from patients 460 and 425 were not available for molecular analyses and the other patients refused to undergo a muscle biopsy. Interestingly, the total* RYR1* mRNA and the total RyR1 protein levels in patients carrying the c.14771_14772insTAGACAGGGTGTTGCTCTGTTGCCCTTCTT (p.F4924_V4925insRQGVALLPFF) variant did not differ significantly from those of the control, which indicates that cells compensate for the low expression of the insertion variant allele by increasing the levels of the other* RYR1* allele.

*RYR1* gene variants have been associated with a variety of clinical and histopathological phenotypes [1, 53]. In particular, some SVs are associated with both CCD and MHS, others only with CCD. Functional characterization of* RYR1* SVs associated with CCD revealed that some mutations cause leaky RyR1 channels [54–56]; others, mainly those located in the pore region, generate an excitation-contraction uncoupled channel [57, 58]. Moreover, MHS/CCD* RYR1* SVs respond to triggering agents with either a reduced or enhanced Ca^2+^ increase in response to 4-CmC or caffeine [54–56, 59]. Furthermore, MHS/CCD mutations located in the N-terminal and the central region of the RyR1 channel cause a greater Ca^2+^-induced Ca^2+^ release than do MHS mutations and enhance RyR1 sensitivity to activating Ca^2+,^ thereby accelerating channel activity [55, 56].

The c.7035C>A (p.S2345R) substitution identified in family NA-36 was associated with a reduction in the Ca^2+^ stores of the ER. Moreover, the cell line from patient 460, who expresses only the* RYR1* c.7035C>A (p.S2345R) allele, shows a percentage of Ca^2+^ release triggered by 4-CmC higher than control and with a dose-response curve shifted to the left (Figure 4(b)), whereas the cell line from patient 461, who expresses the* RYR1* c.7035C>A (p.S2345R) allele at the heterozygous status, shows a percentage of Ca^2+^ release triggered by 4-CmC higher than the control only at a concentration of 1000 *μ*M 4-CmC (Figure 4(b)). Taken together, these data imply that the* RYR1* c.7035C>A (p.S2345R) variant has a dominant effect. The c.7035C>A (p.S2345R) variant has been previously identified in a family with MHS and myopathy [5] and affects an amino acid residue located in the cytoplasmic helical domain 1 (residues 2146-2712) between helices 4b (residues 2325-2340) and 5a (residues 2348-2361) [60]. The p.S2345T substitution, which affects the same codon, has been reported in a family with hyperCKemia and reduced expression of RyR1 [50]. Moreover, the p.S2345R substitution is located close to the previously identified MHS mutations p.Asn2342Ser [25], p.Glu2344Asp [61], and p.Ala2350Thr [24], and MHS/CCD mutation p.Val2346Met [62].

In family NA-39, the compound heterozygosity condition for variants c.9293G>T (p.S3098I) and c.14771_14772insTAGACAGGGTGTTGCTCTGTTGCCCTTCTT (p.F4924_V4925insRQGVALLPFF) causes an increase of RyR1-dependent Ca^2+^ release after 4-CmC administration and does not affect ER Ca^2+^ stores. The insertion variant lies in the S6 transmembrane segment of the RyR1 structure model [37] and is predicted to modify this structure (Supplementary Figures S6 and S7). Although expressed at low levels, the insertion variant could be statistically present with at least one monomer in about 46.8% of tetrameric RyR1 molecules [63] and can influence the behavior of the channels. Nevertheless, a limit of our study is the use of nonmuscle cells for the in vitro characterization of mutants. The c.9293G>T (p.S3098I) substitution was predicted to have a pathological character and affects a residue located in a region involved in the homeostasis of intracellular calcium (amino acids 3770-4007) [64]. On the basis of our experiments, we could not discriminate between the effects of the two variants. Unfortunately, lymphoblastoid cell lines from patients 522 and 530 of family NA-39, carrying only the insertion variant, could not be used for RyR1 functional assays because they rapidly showed signs of replicative senescence, and patients were not available for further blood sampling. Patient 522 has clear signs of myopathy and a clinical picture milder than that of patients 425 and 521, who are composite heterozygous for* RYR1* c.9293G>T (p.S3098I) and c.14771_14772insTAGACAGGGTGTTGCTCTGTTGCCCTTCTT (p.F4924_V4925insRQGVALLPFF). On the basis of our experimental evidence, we might speculate that the* RYR1* c.9293G>T (p.S3098I) variant could exacerbate the myopathic phenotype of these patients. Patient 530, who carries the same* RYR1 *genotype as patient 522, at his last neurological examination did not show signs of myopathy. Accordingly, variable penetrance and phenotypic expression have been reported for* RYR1*-related myopathies [3, 5, 6, 65–68]. The inheritance of* RYR1*-related myopathies is complex, and, moreover, a high phenotypic variability is associated with some* RYR1* SVs, even in patients of the same family with the same SV and in the same individual at different ages. This variable degree of penetrance might arise from differences between individuals with regard to allele mutant stoichiometry within the tetramer, to the presence or absence of modifying genes, or to a combination of both. Exome sequencing analysis could provide additional information on the genetic background of family NA-39. Moreover,* RYR1 *mutations are the most frequent genetic cause of congenital myopathies and it has become increasingly clear that many congenital myopathies are characterized by nonspecific or complex pathological abnormalities rather than a ‘pure' muscle pathology pattern [53].

## Data Availability

The data used to support the findings of this study are available from the corresponding author upon request.

## Abbreviations

ALT: Alanine aminotransferase
CK: Creatine kinase
DHPLC: Denaturing high-performance liquid chromatography
EBV: Epstein-Barr virus
ER: Endoplasmic reticulum
gDNA: Genomic DNA
IVCT: In vitro contracture test
LDH: Lactate dehydrogenase
MH: Malignant hyperthermia
MHN: MH-normal
MHS: Malignant hyperthermia susceptibility
RYR1: Ryanodine receptor type 1
SR: Sarcoplasmic reticulum
SVs: Sequence variants
SEM: Standard error of the mean
SERCA: Sarcoplasmic reticulum calcium ATPase;
S/ER: Sarco/endoplasmic reticulum
TG: Thapsigargin
4-CmC: 4-chloro-m-cresol.

## Supplementary Materials

Supplementary Materials
* Figure S1:* biopsy of the biceps brachii muscle of patient 460 (family NA-36);* Figure S2,* Sequence analysis of* RYR1 *c.7035C>A (p.S2345R) variant in patients 460 and 461;* Figure S3:* results of splicing prediction effect of the* RYR1 *c.4003C>T variant using the Alamut software;* Figure S4:* the ARMS-PCR system;* Figure S5:* sequence analysis of the insertion variant;* Figure S6:* prediction of the local protein secondary structure of wild type RyR1 and of RyR1p.F4924_V4925insRQGVALLPFF;* Figure S7:* hydrophobicity pattern prediction, prediction of the local protein hydrophobicity pattern of wild type RyR1 (A) and of RyR1p.F4924_V4925insRQGVALLPFF;* Figure S8:* resting intracellular calcium concentration in lymphoblastoid cells from patients 460, 461, and 425;* Table 1 S:* oligonucleotide pairs and PCR conditions for the amplification of RYR1 cDNA.

## References

[1] Carsana A. (2014). RYR1-related myopathies and anesthesiological implications. *International Journal of Clinical Anesthesiology*.

[2] Bevilacqua J. A., Monnier N., Bitoun M. (2011). Recessive RYR1 mutations cause unusual congenital myopathy with prominent nuclear internalization and large areas of myofibrillar disorganization. *Neuropathology and Applied Neurobiology*.

[3] Klein A., Lillis S., Munteanu I. (2012). Clinical and genetic findings in a large cohort of patients with ryanodine receptor 1 gene-associated myopathies. *Human Mutation*.

[4] Amburgey K., Bailey A., Hwang J. H. (2013). Genotype-phenotype correlations in recessive RYR1-related myopathies. *Orphanet Journal of Rare Diseases*.

[5] Snoeck M., van Engelen B. G. M., Küsters B. (2015). RYR1-related myopathies: A wide spectrum of phenotypes throughout life. *European Journal of Neurology*.

[6] Samões R., Oliveira J., Taipa R. (2017). RYR1-related myopathies: clinical, histopathologic and genetic heterogeneity among 17 patients from a portuguese tertiary centre. *Journal of Neuromuscular Diseases*.

[7] Amburgey K., McNamara N., Bennett L. R., McCormick M. E., Acsadi G., Dowling J. J. (2011). Prevalence of congenital myopathies in a representative pediatric united states population. *Annals of Neurology*.

[8] Sewry C. A., Jimenez-Mallebrera C., Muntoni F. (2008). Congenital myopathies. *Current Opinion in Neurology*.

[9] Schneiderbanger D., Johannsen S., Roewer N., Schuster F. (2014). Management of malignant hyperthermia: diagnosis and treatment. *Therapeutics and Clinical Risk Management*.

[10] Hopkins P. M., Rüffert H., Snoeck M. M. (2015). European Malignant Hyperthermia Group guidelines for investigation of malignant hyperthermia susceptibility. *British Journal of Anaesthesia*.

[11] Carsana A. (2013). Exercise-induced rhabdomyolysis and stress-induced malignant hyperthermia events, association with malignant hyperthermia susceptibility, and RYR1 gene sequence variations. *The Scientific World Journal*.

[12] Carsana A. (2013). Exertional rhabdomyolysis, RYR1 gene sequence variations and association with malignant hyperthermia susceptibility. *International Journal of Clinical Anesthesiology*.

[13] Weiss R. G., O'Connell K. M. S., Flucher B. E., Allen P. D., Grabner M., Dirksen R. T. (2004). Functional analysis of the R1086H malignant hyperthermia mutation in the DHPR reveals an unexpected influence of the III-IV loop on skeletal muscle EC coupling. *American Journal of Physiology-Cell Physiology*.

[14] Pirone A., Schredelseker J., Tuluc P. (2010). Identification and functional characterization of malignant hyperthermia mutation T1354S in the outer pore of the Cav*α*1S-subunit. *American Journal of Physiology-Cell Physiology*.

[15] Eltit J. M., Bannister R. A., Moua O. (2012). Malignant hyperthermia susceptibility arising from altered resting coupling between the skeletal muscle L-type Ca^2+^ channel and the type 1 ryanodine receptor. *Proceedings of the National Acadamy of Sciences of the United States of America*.

[16] Jungbluth H. (2007). Central core disease. *Orphanet Journal of Rare Diseases*.

[17] Brislin R. P., Theroux M. C. (2013). Core myopathies and malignant hyperthermia susceptibility: A review. *Pediatric Anesthesia*.

[18] Wehner M., Rueffert H., Koenig F., Neuhaus J., Olthoff D. (2002). Increased sensitivity to 4-chloro-m-cresol and caffeine in primary myotubes from malignant hyperthermia susceptible individuals carrying the ryanodine receptor 1 Thr2206Met (C6617T) mutation. *Clinical Genetics*.

[19] Girard T., Treves S., Censier K., Mueller C. R., Zorzato F., Urwyler A. (2002). Phenotyping malignant hyperthermia susceptibility by measuring halothane-induced changes in myoplasmic calcium concentration in cultured human muscle cells. *British Journal of Anaesthesia*.

[20] Brinkmeier H., Krämer J., Krämer R. (1999). Malignant hyperthermia causing Gly2435Arg mutation of the ryanodine receptor facilitates ryanodine-induced calcium release in myotubes. *British Journal of Anaesthesia*.

[21] Yang T., Ta T. A., Pessah I. N., Allen P. D. (2003). Functional defects in six RYR1 mutations associated with malignant hyperthermia and their impact on skeletal E-C coupling. *The Journal of Biological Chemistry*.

[22] Girard T., Cavagna D., Padovan E. (2001). B-lymphocytes from malignant hyperthermia-susceptible patients have an increased sensitivity to skeletal muscle ryanodine receptor activators. *The Journal of Biological Chemistry*.

[23] Tong J., Oyamada H., Demaurex N., Grinstein S., McCarthy T. V., MacLennan D. H. (1997). MacLennan DH (1997) Caffeine and halothane sensitivity of intracellular Ca^2+^ release is altered by 15 calcium release channel (ryanodine receptor) mutations associated with malignant hyperthermia and/or central core disease. *The Journal of Biological Chemistry*.

[24] Sambuughin N., Nelson T. E., Jankovic J. (2001). Identification and functional characterization of a novel ryanodine receptor mutation causing malignant hyperthermia in North American and South American families. *Neuromuscular Disorders*.

[25] Zullo A., Klingler W., De Sarno C. (2009). Functional characterization of ryanodine receptor (RYR1) sequence variants using a metabolic assay in immortalized B-lymphocytes. *Human Mutation*.

[26] Hoppe K., Hack G., Lehmann-Horn F. (2016). Hypermetabolism in B-lymphocytes from malignant hyperthermia susceptible individuals. *Scientific Reports*.

[27] Robinson R. L., Anetseder M. J., Brancadoro V. (2003). Recent advances in the diagnosis of malignant hyperthermia susceptibility: How confident can we be of genetic testing?. *European Journal of Human Genetics*.

[28] Fortunato G., Carsana A., Tinto N., Brancadoro V., Canfora G., Salvatore F. (1999). A case of discordance between genotype and phenotype in a malignant hyperthermia family. *European Journal of Human Genetics*.

[29] Ording H., Brancadoro V., Cozzolino S. (1997). In vitro contracture test for diagnosis of malignant hyperthermia following the protocol of the European MH group: Results of testing patients surviving fulminant MH and unrelated low-risk subjects. *Acta Anaesthesiologica Scandinavica*.

[30] Lek M., Karczewski K. J., Minikel E. V. (2016). Exome Aggregation Consortium. Analysis of protein-coding genetic variation in 60,706 humans. *Nature*.

[31] Neitzel H. (1986). A routine method for the establishment of permanent growing lymphoblastoid cell lines. *Human Genetics*.

[32] Chen H., Li Y., Tollefsbol T. O. (2013). Cell senescence culturing methods. *Methods in Molecular Biology*.

[33] Livak K. J., Schmittgen T. D. (2001). Analysis of relative gene expression data using real-time quantitative PCR and the 2^-ΔΔCt^ method. *Methods*.

[34] Breljak D., Ambriovic-Ristov A., Kapitanovic S., Cavec T., Gabrilovac J. (2005). Comparison of three RT-PCR based methods for relative quantification of mRNA. *Food Technology and Biotechnology*.

[35] Rossi D., Simeoni I., Micheli M. (2002). RyR1 and RyR3 isoforms provide distinct intracellular Ca^2+^ signals in HEK293 cells. *Journal of Cell Science*.

[36] Bailey S., Macardle P. J. (2006). A flow cytometric comparison of Indo-1 to fluo-3 and Fura Red excited with low power lasers for detecting Ca^2+^ flux. *Journal of Immunological Methods*.

[37] Zalk R., Clarke O. B., des Georges A. (2015). Structure of a mammalian ryanodine receptor. *Nature*.

[38] Sei Y., Gallagher K. L., Basile A. S. (1999). Skeletal muscle type ryanodine receptor is involved in calcium signaling in human B lymphocytes. *The Journal of Biological Chemistry*.

[39] Herrmann-Frank A., Richter M., Lehmann-Horn F. (1996). 4-Chloro-m-cresol: A specific tool to distinguish between malignant hyperthermia-susceptible and normal muscle. *Biochemical Pharmacology*.

[40] Tegazzin V., Scutari E., Treves S., Zorzato F. (1996). Chlorocresol an additive to commercial succinylcholine induces contracture of human malignant hyperthermia-susceptible muscles via activation of the ryanodine receptor Ca^2+^ channel. *Anesthesiology*.

[41] Duke A. M., Steele D. S. (1998). Effects of cyclopiazonic acid on Ca^2+^ regulation by the sarcoplasmic reticulum in saponin-permeabilized skeletal muscle fibers. *Pflügers Archiv - European Journal of Physiology*.

[42] Thastrup O., Cullen P. J., Drobak B. K., Hanley M. R., Dawson A. P. (1990). Thapsigargin a tumor promoter discharges intracellular Ca^2+^ stores by specific inhibition of the endoplasmic reticulum Ca^2+^-ATPase. *Proceedings of the National Acadamy of Sciences of the United States of America*.

[43] Tilgen N., Zorzato F., Halliger-Keller B. (2001). Identification of four novel mutations in the C-terminal membrane spanning domain of the ryanodine receptor 1: Association with central core disease and alteration of calcium homeostasis. *Human Molecular Genetics*.

[44] Johannsen S., Treves S., Müller C. R. (2016). Functional characterization of the RYR1 mutation p.Arg4737Trp associated with susceptibility to malignant hyperthermia. *Neuromuscular Disorders*.

[45] Kurosaki T., Myers J. R., Maquat L. E. (2019). Defining nonsense-mediated mRNA decay intermediates in human cells. *Methods*.

[46] Attali R., Aharoni S., Treves S. (2013). Variable myopathic presentation in a single family with novel skeletal RYR1 mutation. *PLoS ONE*.

[47] Grievink H., Stowell K. M. (2010). Allele-specific differences in ryanodine receptor 1 mRNA expression levels may contribute to phenotypic variability in malignant hyperthermia. *Orphanet Journal of Rare Diseases*.

[48] Kraeva N., Heytens L., Jungbluth H. (2015). Compound RYR1 heterozygosity resulting in a complex phenotype of malignant hyperthermia susceptibility and a core myopathy. *Neuromuscular Disorders*.

[49] Rokach O., Sekulic-Jablanovic M., Voermans N. (2015). Epigenetic changes as a common trigger of muscle weakness in congenital myopathies. *Human Molecular Genetics*.

[50] Sano K., Miura S., Fujiwara T. (2015). A novel missense mutation of RYR1 in familial idiopathic hyper CK-emia. *Journal of the Neurological Sciences*.

[51] Zhou H., Brockington M., Jungbluth H. (2006). Epigenetic allele silencing unveils recessive RYR1 mutations in core myopathies. *American Journal of Human Genetics*.

[52] Zhou H., Jungbluth H., Sewry C. A. (2007). Molecular mechanisms and phenotypic variation in RYR1-related congenital myopathies. *Brain*.

[53] Jungbluth H., Treves S., Zorzato F. (2018). Congenital myopathies: Disorders of excitation-contraction coupling and muscle contraction. *Nature Reviews Neurology*.

[54] Brini M., Manni S., Pierobon N. (2005). Ca2+ signaling in HEK-293 and skeletal muscle cells expressing recombinant ryanodine receptors harboring malignant hyperthermia and central core disease mutations. *The Journal of Biological Chemistry*.

[55] Murayama T., Kurebayashi N., Ogawa H. (2016). Genotype-phenotype correlations of malignant hyperthermia and central core disease Mutations in the central region of the RYR1 channel. *Human Mutation*.

[56] Murayama T., Kurebayashi N., Yamazawa T. (2015). Divergent activity profiles of type 1 ryanodine receptor channels carrying malignant hyperthermia and central core disease mutations in the amino-terminal region. *PLoS ONE*.

[57] Avila G., O'Brien J. J., Dirksen R. T. (2001). Excitation-contraction uncoupling by a human central core disease mutation in the ryanodine receptor. *Proceedings of the National Acadamy of Sciences of the United States of America*.

[58] Avila G., O'Connell K. M., Dirksen R. T. (2003). The pore region of the skeletal muscle ryanodine receptor is a primary locus for excitation-contraction uncoupling in central core disease. *The Journal of General Physiology*.

[59] Ducreux S., Zorzato F., Müller C. (2004). Effect of ryanodine receptor mutations on interleukin-6 release and intracellular calcium homeostasis in human myotubes from malignant hyperthermia-susceptible individuals and patients affected by central core disease. *The Journal of Biological Chemistry*.

[60] Yan Z., Bai X.-C., Yan C. (2015). Structure of the rabbit ryanodine receptor RyR1 at near-atomic resolution. *Nature*.

[61] Monnier N., Kozak-Ribbens G., Krivosic-Horber R. (2005). Correlations between genotype and pharmacological histological functional and clinical phenotypes in malignant hyperthermia susceptibility. *Human Mutation*.

[62] Shepherd S., Ellis F., Halsall J., Hopkins P., Robinson R. (2004). RYR1 mutations in UK central core disease patients: more than just the C-terminal transmembrane region of the RYR1 gene. *Journal of Medical Genetics*.

[63] Chan W. M., Siu W. Y., Lau A., Poon R. Y. C. (2004). How many mutant p53 molecules are needed to inactivate a tetramer?. *Molecular and Cellular Biology*.

[64] Chen Y., Xue S., Zou J., Lopez J. R., Yang J. J., Perez C. F. (2014). Myoplasmic resting Ca2+ regulation by ryanodine receptors is under the control of a novel Ca2+-binding region of the receptor. *Biochemical Journal*.

[65] Quinlivan R. M., Muller C. R., Davis M. (2003). Central Core disease: clinical, pathological and genetic features. *Archives of Disease in Childhood*.

[66] Dowling J. J., Lillis S., Amburgey K. (2011). King-Denborough syndrome with and without mutations in the skeletal muscle ryanodine receptor (RYR1) gene. *Neuromuscular Disorders*.

[67] Kraeva N., Sapa A., Dowling J. J., Riazi S. (2017). Malignant hyperthermia susceptibility in patients with exertional rhabdomyolysis: a retrospective cohort study and updated systematic review. *Canadian Journal of Anesthesia*.

[68] Kraeva N., Heytens L., Jungbluth H. (2015). Compound RYR1 heterozygosity resulting in a complex phenotype of malignant hyperthermia susceptibility and a core myopathy. *Neuromuscular Disorders*.

