# Supplementary figures and images for: RYR1 Sequence Variants in Myopathies: Expression and Functional Studies in Two Families
Source: Biomed Res Int. 2019 Apr 21;2019:7638946. doi: 10.1155/2019/7638946 (PMC6500691; doi:10.1155/2019/7638946)

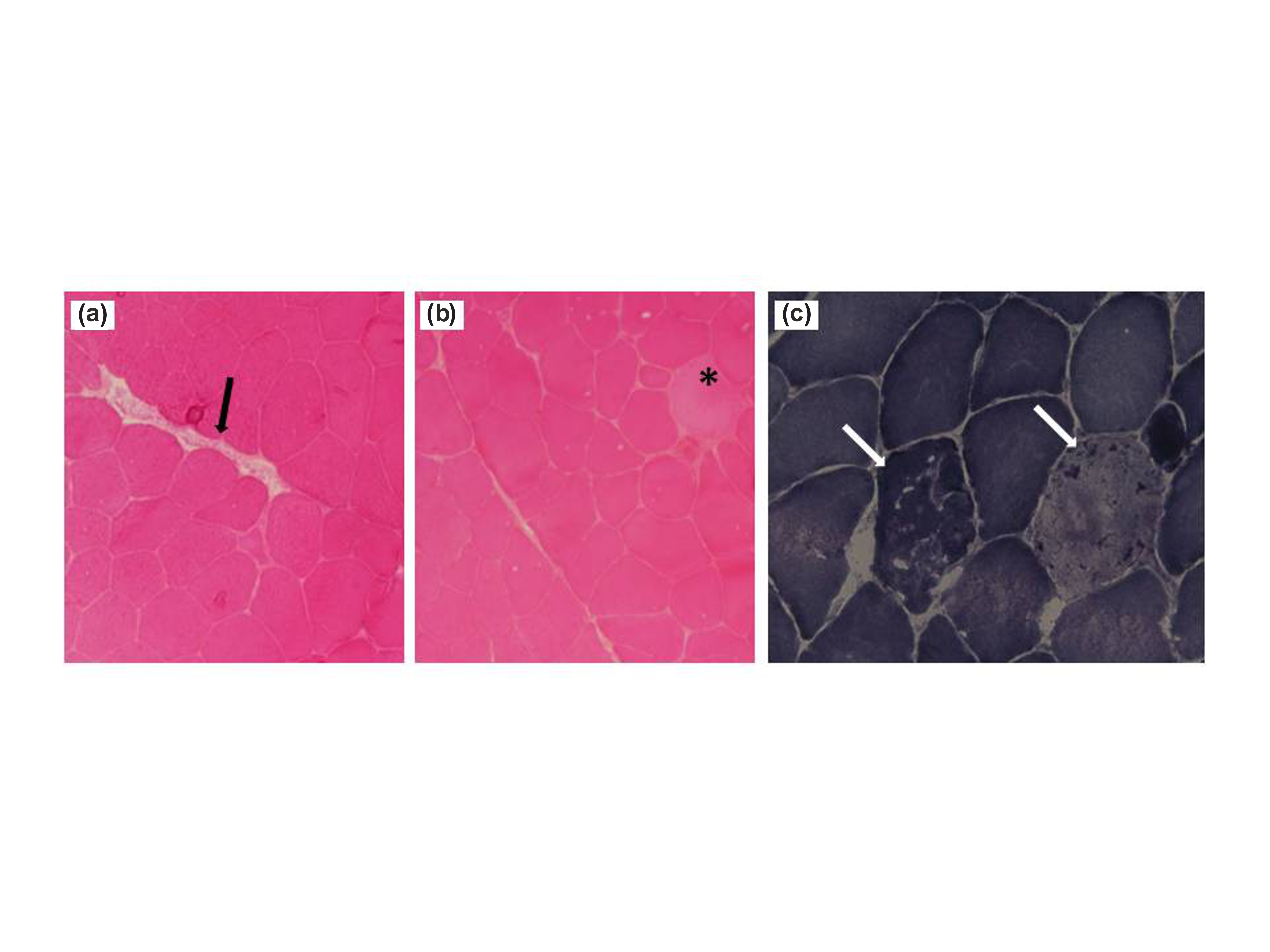

Supplement: Supplementary Materials — Figure S1: biopsy of the biceps brachii muscle of patient 460 (family NA-36); Figure S2, Sequence analysis of RYR1 c.7035C>A (p.S2345R) variant in patients 460 and 461; Figure S3: results of splicing prediction effect of the RYR1 c.4003C>T variant using the Alamut software; Figure S4: the ARMS-PCR system; Figure S5: sequence analysis of the insertion variant; Figure S6: prediction of the local protein secondary structure of wild type RyR1 and of RyR1p.F4924_V4925insRQGVALLPFF; Figure S7: hydrophobicity pattern prediction, prediction of the local protein hydrophobicity pattern of wild type RyR1 (A) and of RyR1p.F4924_V4925insRQGVALLPFF; Figure S8: resting intracellular calcium concentration in lymphoblastoid cells from patients 460, 461, and 425; Table 1 S: oligonucleotide pairs and PCR conditions for the amplification of RYR1 cDNA. [file 7638946.f1.zip › Supplementary Figure S1.tif]

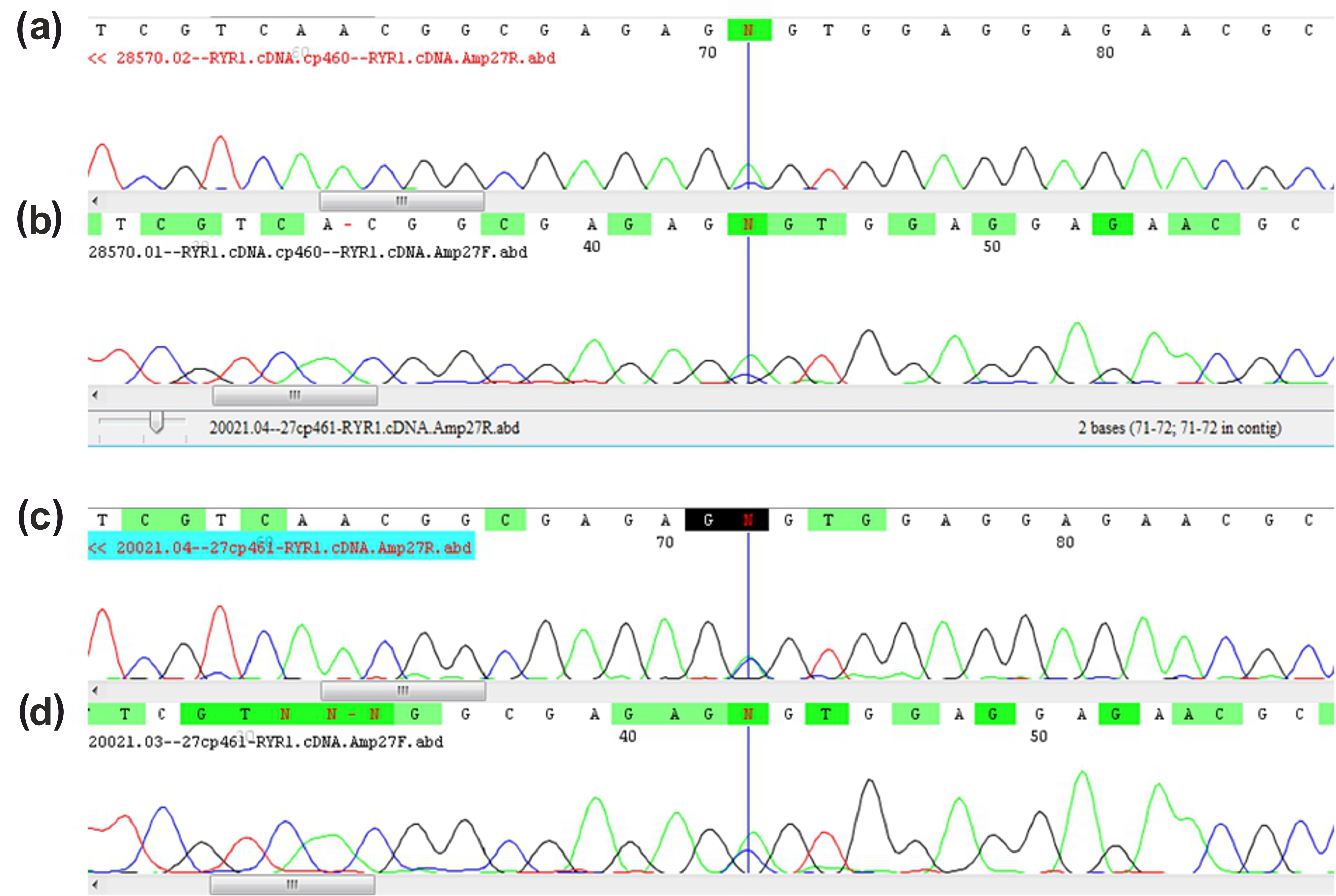

Supplement: Supplementary Materials — Figure S1: biopsy of the biceps brachii muscle of patient 460 (family NA-36); Figure S2, Sequence analysis of RYR1 c.7035C>A (p.S2345R) variant in patients 460 and 461; Figure S3: results of splicing prediction effect of the RYR1 c.4003C>T variant using the Alamut software; Figure S4: the ARMS-PCR system; Figure S5: sequence analysis of the insertion variant; Figure S6: prediction of the local protein secondary structure of wild type RyR1 and of RyR1p.F4924_V4925insRQGVALLPFF; Figure S7: hydrophobicity pattern prediction, prediction of the local protein hydrophobicity pattern of wild type RyR1 (A) and of RyR1p.F4924_V4925insRQGVALLPFF; Figure S8: resting intracellular calcium concentration in lymphoblastoid cells from patients 460, 461, and 425; Table 1 S: oligonucleotide pairs and PCR conditions for the amplification of RYR1 cDNA. [file 7638946.f1.zip › Supplementary Figure S2.tif]

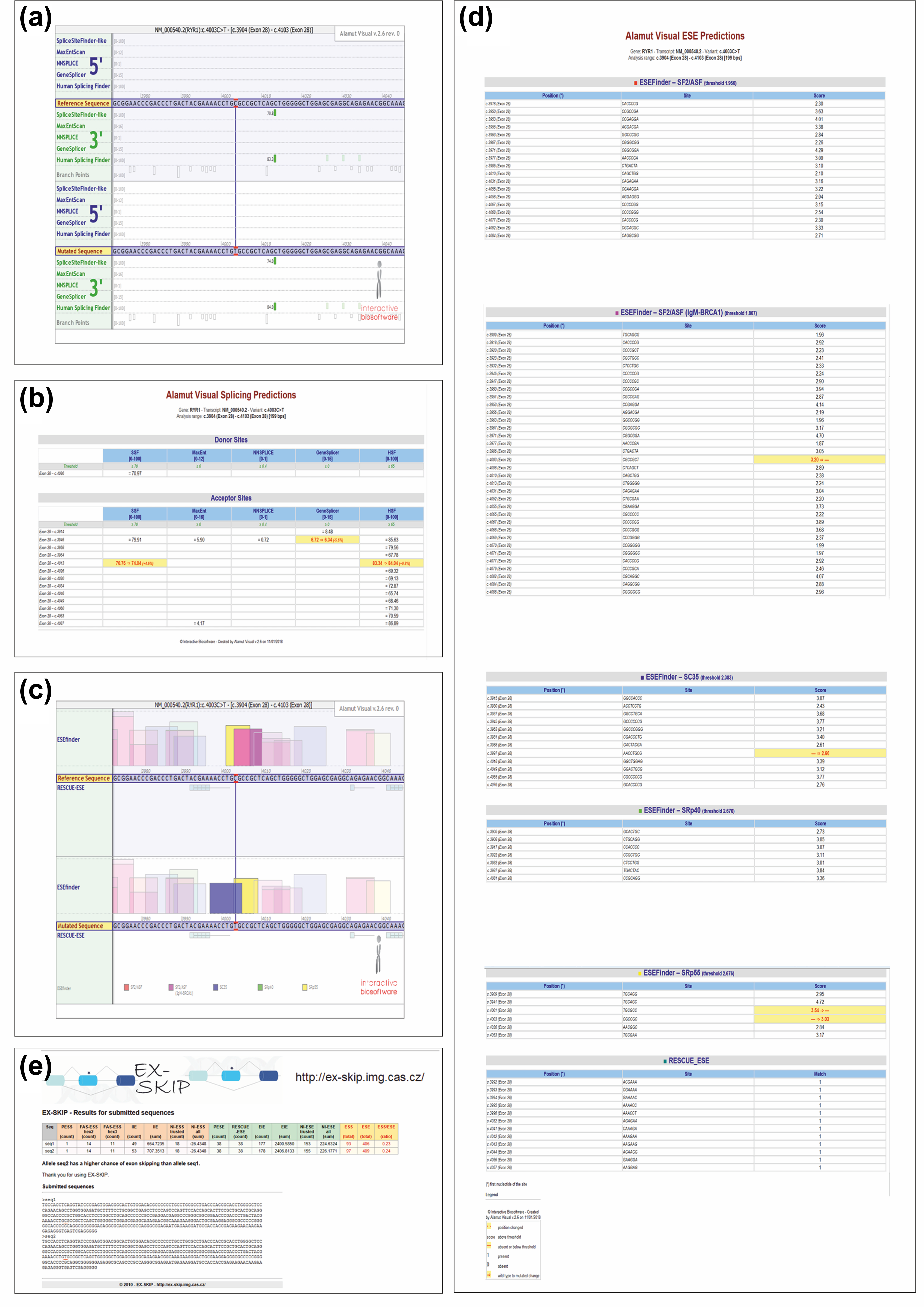

Supplement: Supplementary Materials — Figure S1: biopsy of the biceps brachii muscle of patient 460 (family NA-36); Figure S2, Sequence analysis of RYR1 c.7035C>A (p.S2345R) variant in patients 460 and 461; Figure S3: results of splicing prediction effect of the RYR1 c.4003C>T variant using the Alamut software; Figure S4: the ARMS-PCR system; Figure S5: sequence analysis of the insertion variant; Figure S6: prediction of the local protein secondary structure of wild type RyR1 and of RyR1p.F4924_V4925insRQGVALLPFF; Figure S7: hydrophobicity pattern prediction, prediction of the local protein hydrophobicity pattern of wild type RyR1 (A) and of RyR1p.F4924_V4925insRQGVALLPFF; Figure S8: resting intracellular calcium concentration in lymphoblastoid cells from patients 460, 461, and 425; Table 1 S: oligonucleotide pairs and PCR conditions for the amplification of RYR1 cDNA. [file 7638946.f1.zip › Supplementary Figure S3.tif]

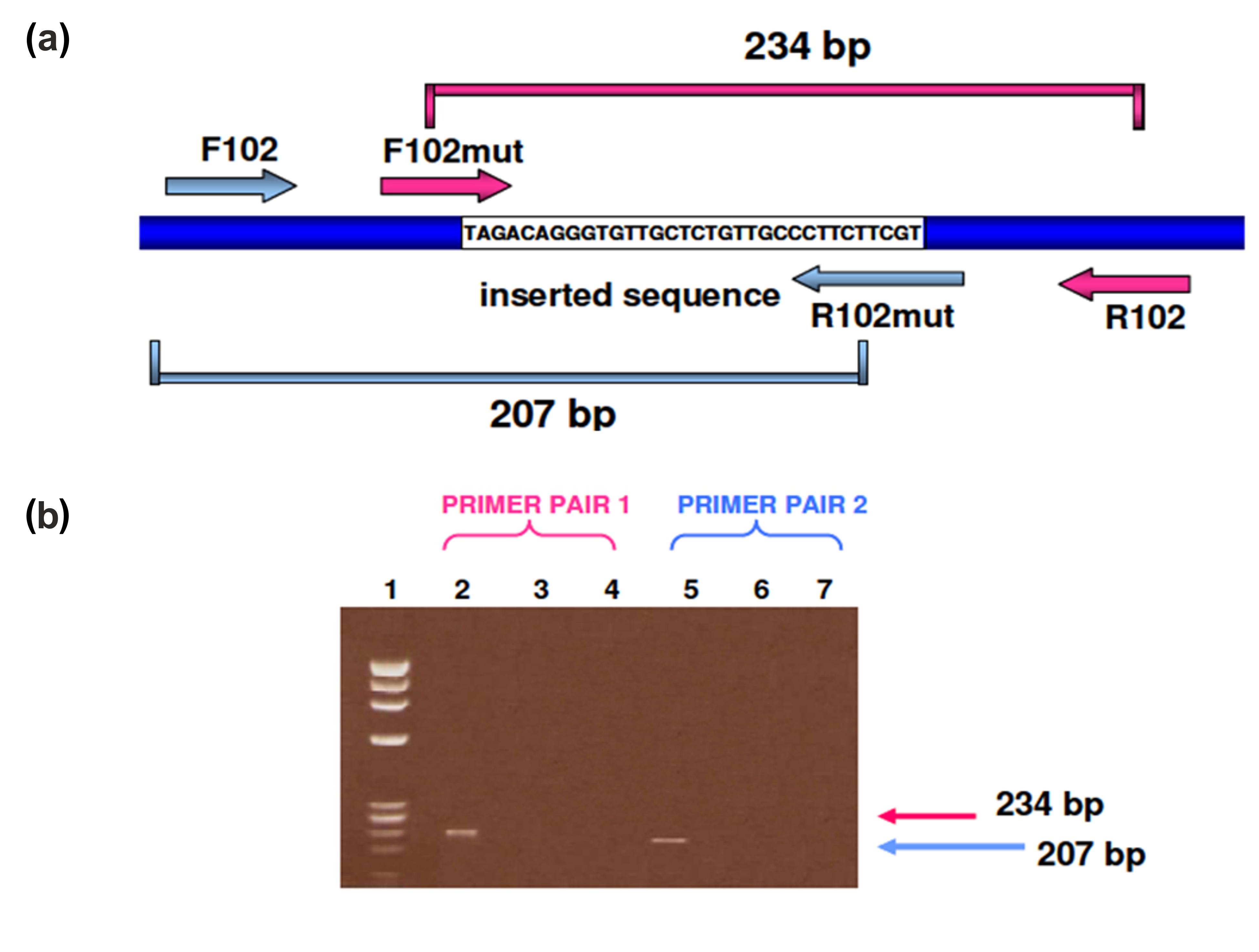

Supplement: Supplementary Materials — Figure S1: biopsy of the biceps brachii muscle of patient 460 (family NA-36); Figure S2, Sequence analysis of RYR1 c.7035C>A (p.S2345R) variant in patients 460 and 461; Figure S3: results of splicing prediction effect of the RYR1 c.4003C>T variant using the Alamut software; Figure S4: the ARMS-PCR system; Figure S5: sequence analysis of the insertion variant; Figure S6: prediction of the local protein secondary structure of wild type RyR1 and of RyR1p.F4924_V4925insRQGVALLPFF; Figure S7: hydrophobicity pattern prediction, prediction of the local protein hydrophobicity pattern of wild type RyR1 (A) and of RyR1p.F4924_V4925insRQGVALLPFF; Figure S8: resting intracellular calcium concentration in lymphoblastoid cells from patients 460, 461, and 425; Table 1 S: oligonucleotide pairs and PCR conditions for the amplification of RYR1 cDNA. [file 7638946.f1.zip › Supplementary Figure S4.tif]

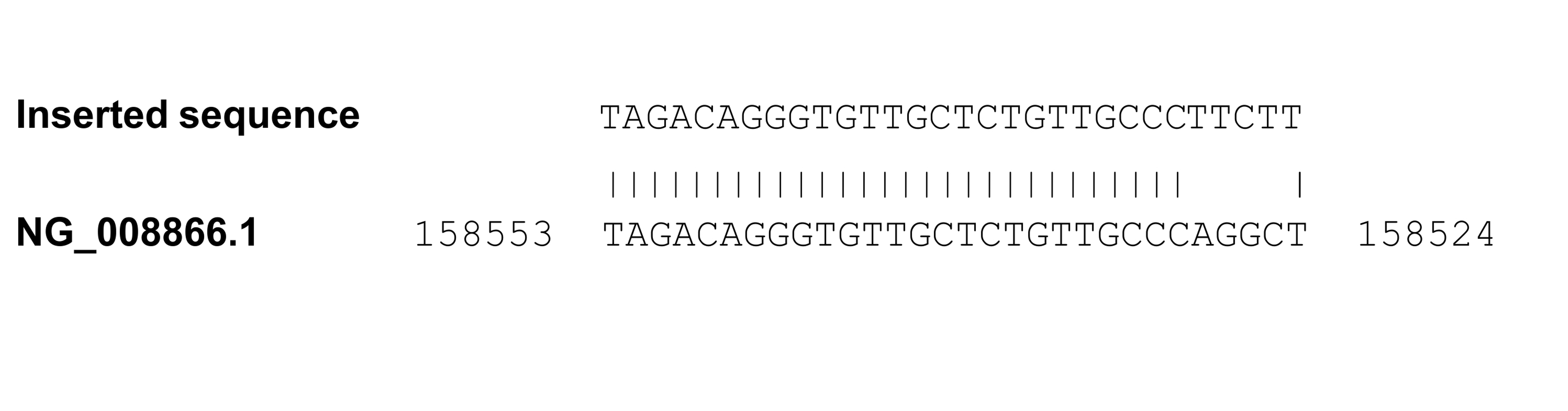

Supplement: Supplementary Materials — Figure S1: biopsy of the biceps brachii muscle of patient 460 (family NA-36); Figure S2, Sequence analysis of RYR1 c.7035C>A (p.S2345R) variant in patients 460 and 461; Figure S3: results of splicing prediction effect of the RYR1 c.4003C>T variant using the Alamut software; Figure S4: the ARMS-PCR system; Figure S5: sequence analysis of the insertion variant; Figure S6: prediction of the local protein secondary structure of wild type RyR1 and of RyR1p.F4924_V4925insRQGVALLPFF; Figure S7: hydrophobicity pattern prediction, prediction of the local protein hydrophobicity pattern of wild type RyR1 (A) and of RyR1p.F4924_V4925insRQGVALLPFF; Figure S8: resting intracellular calcium concentration in lymphoblastoid cells from patients 460, 461, and 425; Table 1 S: oligonucleotide pairs and PCR conditions for the amplification of RYR1 cDNA. [file 7638946.f1.zip › Supplementary Figure S5.tif]

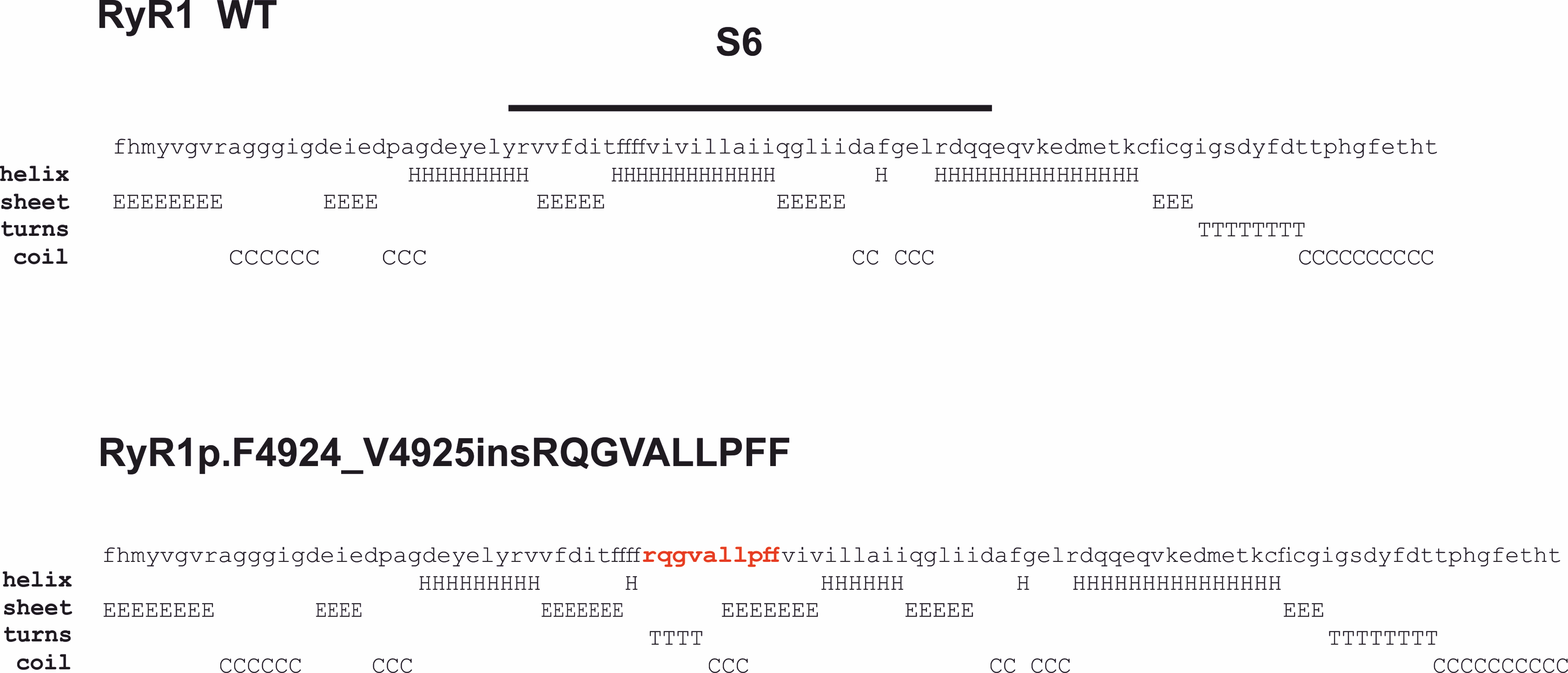

Supplement: Supplementary Materials — Figure S1: biopsy of the biceps brachii muscle of patient 460 (family NA-36); Figure S2, Sequence analysis of RYR1 c.7035C>A (p.S2345R) variant in patients 460 and 461; Figure S3: results of splicing prediction effect of the RYR1 c.4003C>T variant using the Alamut software; Figure S4: the ARMS-PCR system; Figure S5: sequence analysis of the insertion variant; Figure S6: prediction of the local protein secondary structure of wild type RyR1 and of RyR1p.F4924_V4925insRQGVALLPFF; Figure S7: hydrophobicity pattern prediction, prediction of the local protein hydrophobicity pattern of wild type RyR1 (A) and of RyR1p.F4924_V4925insRQGVALLPFF; Figure S8: resting intracellular calcium concentration in lymphoblastoid cells from patients 460, 461, and 425; Table 1 S: oligonucleotide pairs and PCR conditions for the amplification of RYR1 cDNA. [file 7638946.f1.zip › Supplementary Figure S6.tif]

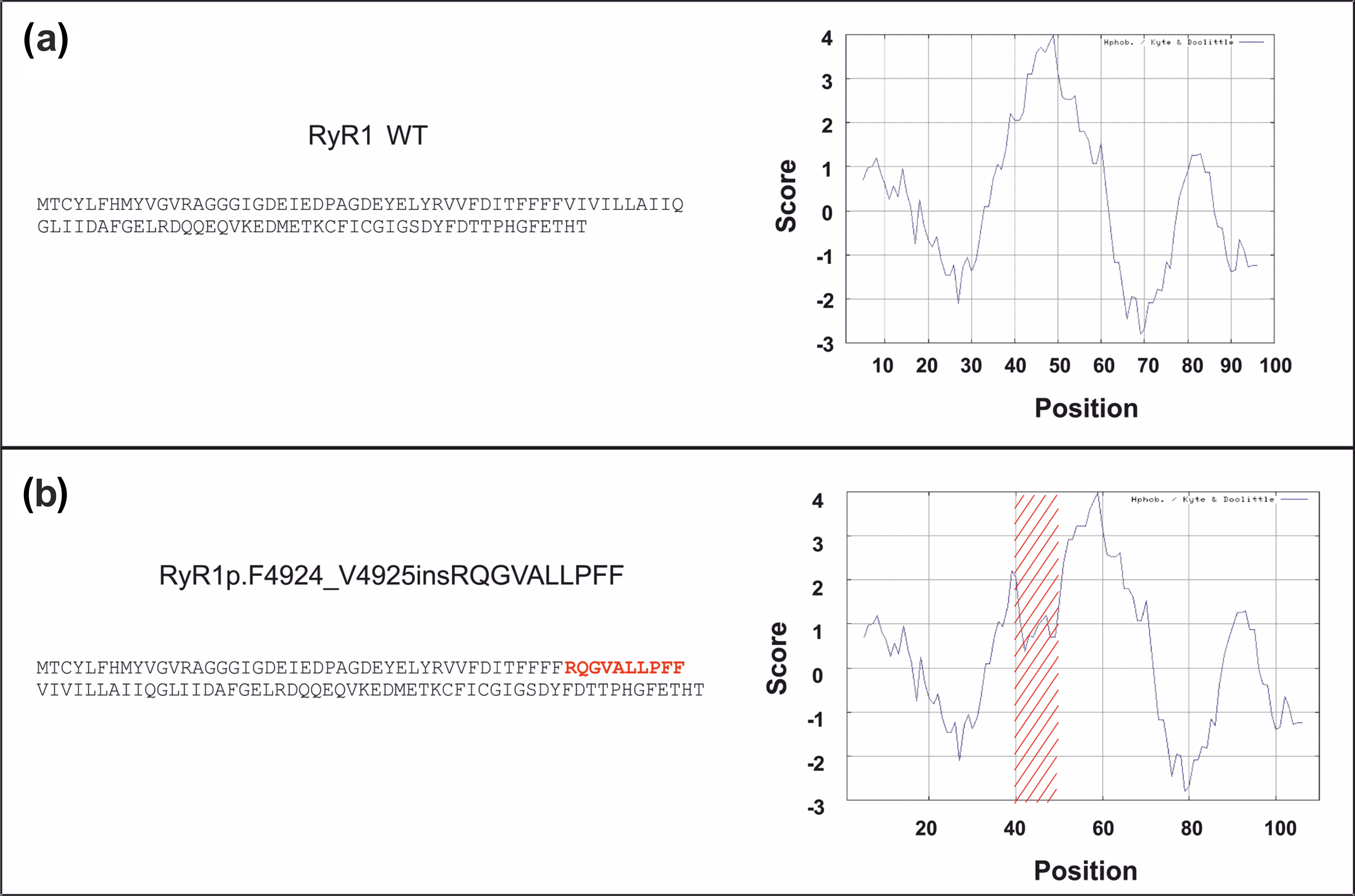

Supplement: Supplementary Materials — Figure S1: biopsy of the biceps brachii muscle of patient 460 (family NA-36); Figure S2, Sequence analysis of RYR1 c.7035C>A (p.S2345R) variant in patients 460 and 461; Figure S3: results of splicing prediction effect of the RYR1 c.4003C>T variant using the Alamut software; Figure S4: the ARMS-PCR system; Figure S5: sequence analysis of the insertion variant; Figure S6: prediction of the local protein secondary structure of wild type RyR1 and of RyR1p.F4924_V4925insRQGVALLPFF; Figure S7: hydrophobicity pattern prediction, prediction of the local protein hydrophobicity pattern of wild type RyR1 (A) and of RyR1p.F4924_V4925insRQGVALLPFF; Figure S8: resting intracellular calcium concentration in lymphoblastoid cells from patients 460, 461, and 425; Table 1 S: oligonucleotide pairs and PCR conditions for the amplification of RYR1 cDNA. [file 7638946.f1.zip › Supplementary Figure S7.tif]

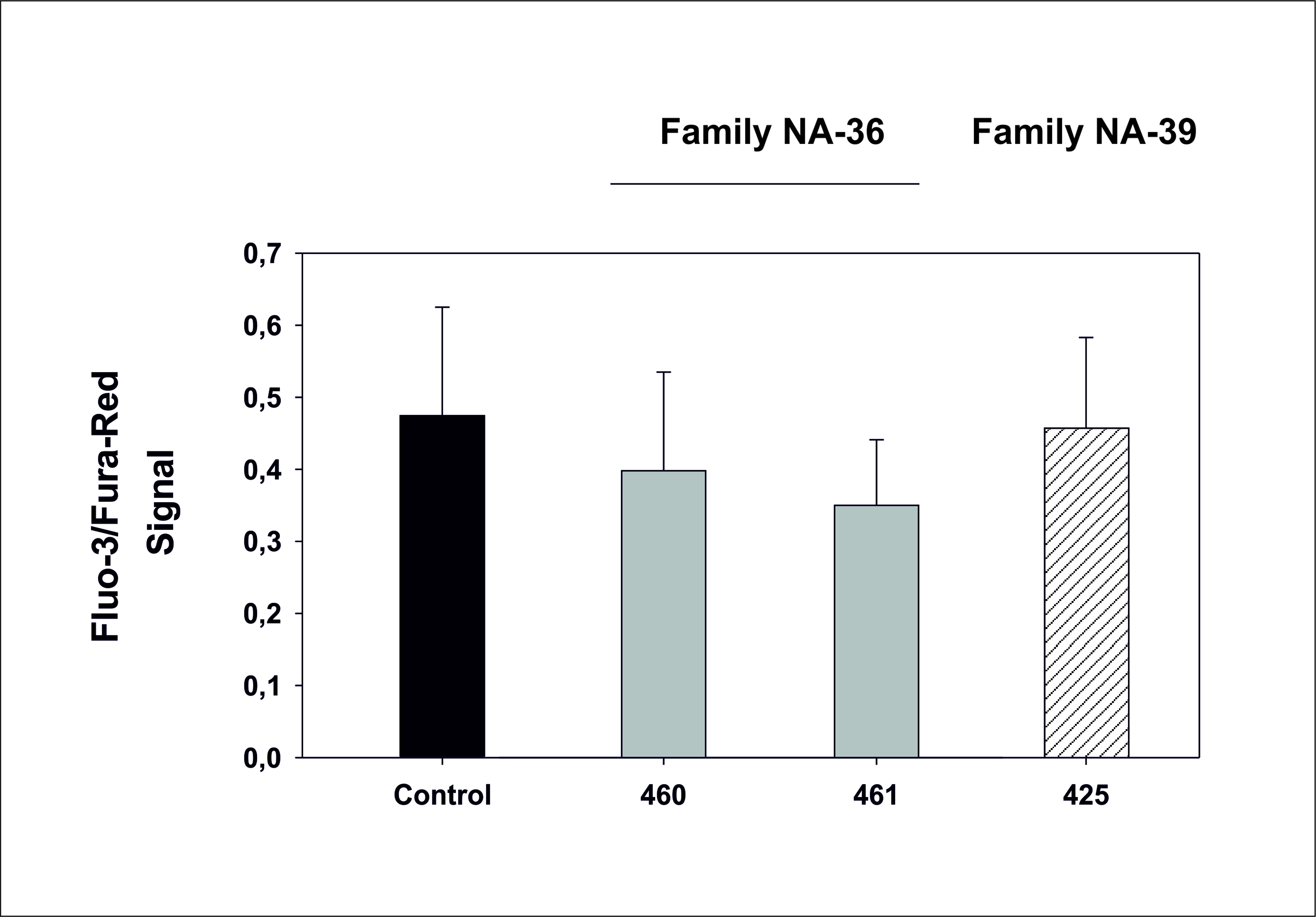

Supplement: Supplementary Materials — Figure S1: biopsy of the biceps brachii muscle of patient 460 (family NA-36); Figure S2, Sequence analysis of RYR1 c.7035C>A (p.S2345R) variant in patients 460 and 461; Figure S3: results of splicing prediction effect of the RYR1 c.4003C>T variant using the Alamut software; Figure S4: the ARMS-PCR system; Figure S5: sequence analysis of the insertion variant; Figure S6: prediction of the local protein secondary structure of wild type RyR1 and of RyR1p.F4924_V4925insRQGVALLPFF; Figure S7: hydrophobicity pattern prediction, prediction of the local protein hydrophobicity pattern of wild type RyR1 (A) and of RyR1p.F4924_V4925insRQGVALLPFF; Figure S8: resting intracellular calcium concentration in lymphoblastoid cells from patients 460, 461, and 425; Table 1 S: oligonucleotide pairs and PCR conditions for the amplification of RYR1 cDNA. [file 7638946.f1.zip › Supplementary Figure S8.tif]
