# Supplementary material for: RYR1 Sequence Variants in Myopathies: Expression and Functional Studies in Two Families
Source: Biomed Res Int. 2019 Apr 21;2019:7638946. doi: 10.1155/2019/7638946 (PMC6500691; doi:10.1155/2019/7638946)
Supplement: Supplementary Materials — Figure S1: biopsy of the biceps brachii muscle of patient 460 (family NA-36); Figure S2, Sequence analysis of RYR1 c.7035C>A (p.S2345R) variant in patients 460 and 461; Figure S3: results of splicing prediction effect of the RYR1 c.4003C>T variant using the Alamut software; Figure S4: the ARMS-PCR system; Figure S5: sequence analysis of the insertion variant; Figure S6: prediction of the local protein secondary structure of wild type RyR1 and of RyR1p.F4924_V4925insRQGVALLPFF; Figure S7: hydrophobicity pattern prediction, prediction of the local protein hydrophobicity pattern of wild type RyR1 (A) and of RyR1p.F4924_V4925insRQGVALLPFF; Figure S8: resting intracellular calcium concentration in lymphoblastoid cells from patients 460, 461, and 425; Table 1 S: oligonucleotide pairs and PCR conditions for the amplification of RYR1 cDNA. [file 7638946.f1.zip › Legends to supplementary figures.doc]

**Legends to supplementary figures**

**Supplementary Figure S1:** Biopsy of the biceps brachii muscle of patient 460 (family NA-36). Staining with hematoxylin/eosin ((a) and (b)) and NADH-tetrazolium reductase (c). Variability of fiber morphology, with 30% of rounded atrophic fibers and about 10% increase in connective tissue, compared to normal value, were observed (black arrow in panel (a)). Moreover, 5% of fibers were represented by ghost fibers (asterisk in panel (b)) and 10% by degenerating fibers (white arrows in panel (c)).

**Supplementary Figure S2:** Sequence analysis of *RYR1* c.7035C>A (p.S2345R) variant on both strands (reverse and forward) in patients 460 ((a) and (b)) and 461 ((c) and (d)) (cDNA amplification reaction 27, see Supplementary Table S1).

**Supplementary Figure S3:** Results of splicing prediction effect of the *RYR1* c.4003C>T variant using the Alamut software. (a) graphical representation of splicing signals prediction. (b) analytical report of splicing signals prediction. (c) graphical representation of ESE reports. (d) analytical report of ESE finder and E. EX-SKIP analysis of *RYR1* exon 28 without (seq1) or with (seq 2) the c.4003C>T variant. The substituted nucleotide is underlined.

**Supplementary Figure S4:** The ARMS-PCR system. (a) Primer design and scheme of PCR products. Primer pair 1: F102mut 5'-CGACATCACCTTCTTCTTCTT**TAG** and R102 5'-GAGGCCCCGAGAGGTGACTTGCC. Primer pair 2: F102 5’-CGAATGAATGAGTGACCAGTGTGC and R102mut 5'-GATGACGATG**ACGAAGAAGGG**. Primer sequences that specifically anneal with the inserted sequence are underlined. (b) Agarose gel electrophoresis of ARMS PCR products. Lane 1: DNA molecular weight marker IX (Roche); lanes 2, 5: PCR products from the patient 425; lanes 3, 6: PCR products from wild type DNA; lanes 4, 7: mock. Specific amplification products are present only in patient 425.

**Supplementary Figure S5:** Sequence analysis of the insertion variant. The first 25 nt of the c.14771_14772insTAGACAGGGTGTTGCTCTGTTGCCCTTCTT sequence, segregating in Family NA-39, corresponds to an inverted and complementary sequence (158553-158529) located at the *RYR1* intron 105. Blastn (https://blast.ncbi.nlm.nih.gov).

**Supplementary Figure S6:** Secondary structure prediction. Prediction of the local protein secondary structure of wild type RyR1 and of RyR1p.F4924_V4925insRQGVALLPFF using the Emboss Garnier software (www.bioinformatics.nl/cgi-bin/emboss/garnier‎). The S6 transmembrane segment (aa 4914-4937) of the RYR1 structure model proposed by Zalk et al 2015 is indicated.

**Supplementary Figure S7:** Hydrophobicity pattern prediction. Prediction of the local protein hydrophobicity pattern of wild type RyR1 (a) and of RyR1p.F4924_V4925insRQGVALLPFF (b) using the Hydrophobicity Kyte & Doolittle plot (http://web.expasy.org/protscale). The inserted sequence and the position of the insertion are highlighted in red.

**Supplementary Figure S8:** Resting intracellular calcium concentration in lymphoblastoid cells from patients 460, 461 and 425 expressing the *RYR1* c.7035C>A (p.S2345R ), the *RYR1* wt/*RYR1* c.7035C>A (p.S2345R) and the *RYR1* c.9293G>T (p.S3098I)/*RYR1* c.14771_14772insTAGACAGGGTGTTGCTCTGTTGCCCTTCTT (p.F4924_V4925insRQGVALLPFF) variant alleles, respectively, and in two control cell lines. Fluo-3, Fura-red and Fluo-3/Fura-red emission signals were recorded for 2 min before stimulation to obtain baseline fluorescence values. Data from the two control cell lines have been averaged. Data are presented as mean ± standard error of the mean (SEM) of at least six experiments. Statistical analysis was performed by one-way analysis of variance and by the two-tailed Student’s t-test (p=0.285, 0.056 and 0.788 for lymphoblastoid cells from patients 460, 461 and 425, respectively, versus control).
