# Supplementary material for: RYR1 Sequence Variants in Myopathies: Expression and Functional Studies in Two Families
Source: Biomed Res Int. 2019 Apr 21;2019:7638946. doi: 10.1155/2019/7638946 (PMC6500691; doi:10.1155/2019/7638946)
Supplement: Supplementary Materials — Figure S1: biopsy of the biceps brachii muscle of patient 460 (family NA-36); Figure S2, Sequence analysis of RYR1 c.7035C>A (p.S2345R) variant in patients 460 and 461; Figure S3: results of splicing prediction effect of the RYR1 c.4003C>T variant using the Alamut software; Figure S4: the ARMS-PCR system; Figure S5: sequence analysis of the insertion variant; Figure S6: prediction of the local protein secondary structure of wild type RyR1 and of RyR1p.F4924_V4925insRQGVALLPFF; Figure S7: hydrophobicity pattern prediction, prediction of the local protein hydrophobicity pattern of wild type RyR1 (A) and of RyR1p.F4924_V4925insRQGVALLPFF; Figure S8: resting intracellular calcium concentration in lymphoblastoid cells from patients 460, 461, and 425; Table 1 S: oligonucleotide pairs and PCR conditions for the amplification of RYR1 cDNA. [file 7638946.f1.zip › Supplementary Table_1S.doc]

**Supplementary Table 1S Oligonucleotide pairs and PCR conditions for the amplification of *RYR1* cDNA. The nucleotide numbering was based on the human *RYR1* transcript reference sequence NM_000540.2**

|  | **Forward primer** | **Reverse primer** | **Ta**  **(°C)** | **MgCl_2_**  **(mM)** | **Enhancer** | **Amplified region** | **Amplicon**  **(bp)** |
| --- | --- | --- | --- | --- | --- | --- | --- |
| 1. | TGTTCCCCGACCTCAGACCCT | CGCCAGCCTCCACCGTGTTAG | 54 | 1.5 |  | c.-41-265 | 306 |
| 2. | CTGGCCATCTGTTGCTTCGTC | AGCCTCTCCTGTTGCGTCCTC | 58 | 1.5 |  | c.181-432 | 252 |
| 3. | CAGGAGGACGCAACAGGAGAG | AGCAGGGGAAATGGTCAGACA | 58 | 1.5 |  | c.410-705 | 297 |
| 4. | CACGTCCTCCGCCTCTTTCAT | CTGCACGAAGCACAGTGACTC | 56 | 1.5 |  | c.649-1044 | 396 |
| 5. | TCCAAGGAGAAGCTGGATGTG | GCCATTGGTGCTGTGGATCAT | 54 | 1.5 |  | c.952-1224 | 273 |
| 6. | GGACGACGCACTGTCGCTGA | CCAGCCAGTCCAAGTTTGTGGAGA | 50 | 1.5 |  | c.1149-1639 | 491 |
| 7. | TCCTAGCTTCTCTAATCCGTGGCAATC | GGGGCGGATGCTGGTGACAT | 59 | 1.5 |  | c.1571-1935 | 365 |
| 8. | TCATCCAGGAGAATCACATCA | AGTCACCTCGTCCACCATCAC | 50 | 1.5 |  | c.1727-2013 | 287 |
| 9. | ACCAGCATCCGCCCCAACAT | ACGCTGAGGTCCAGGCAGCA | 59 | 1.5 |  | c.1921-2252 | 332 |
| 10. | CACCTCCTGGCCCCTGAAGAC | GGGCACGAAGTCGGTGTGTGA | 61 | 1.5 |  | c.2203-2556 | 354 |
| 11. | CTCGAGAGCGACTCCATCTTG | GTTGTCCTCCGCCTTCTCATC | 57 | 1.5 |  | c.2447-2844 | 398 |
| 12. | TCTGGGGAGACGCTCAAGACT | CTTCATCCAGCAGGCGGTAGG | 58 | 1.5 |  | c.2770-3085 | 316 |
| 13. | TACAGCGCAGTGCAGGACATC | CGGTGCCCATTGAAGACATAG | 58 | 1.5 |  | c.3016-3380 | 365 |
| 14. | GCAGTCACCACAGGCGAGATG | ATGGCAAAGAACCTCAGAGAG | 54 | 1.5 |  | c.3277-3644 | 368 |
| 15. | GGCCAGGTGGGTCATCTGAAC | GCGGCGCAGGTTTTCGTAGTC | 58 | 1.5 |  | c.3586-4008 | 423 |
| 16. | GGAGATGCTTTTCCTGCGGCTGA | CACACGCAGCTGGGCTCCTG | 60 | 1 | DMSO 10% | c.3849-4343 | 495 |
| 17. | ACCCCGAGATCATCCTCAACA | CCATTGGCTGTAAAGGTCATT | 56 | 1.5 |  | c.4262-4592 | 331 |
| 18. | TCAGCCACACGGACCTTGTCA | GGCATGCGGCTCCAGGACACT | 56 | 2 |  | c.4520-4800 | 301 |
| 19. | CGCGGCTGGAGATGCAGATG | GCGGCAGGCACTTTCGAGGT | 59 | 1.5 |  | c.4775-5172 | 398 |
| 20. | CACTGCGCGCAGGCTACTATG | GCCTCCCCCAGCATCCTCAGT | 54 | 1.5 |  | c.5114-5453 | 340 |
| 21. | AGGCCCCCGCATCATTTCTC | TCCTCATCTTCCTCTTCACCCTCTTC | 55 | 1.5 |  | c.5314-5669 | 356 |
| 22. | ACCCTGCTGGTGATGGGCAT | GGCCTGGAGCTTGTCCACAT | 52 | 1.5 |  | c.5539-5910 | 375 |
| 23. | AGAAAGAAGAAGGCTTGGAGG | CGGCTGCCCAGGGTGGTCTC | 56 | 1.5 |  | c.5880-6183 | 303 |
| 24. | CATCAAGACCTGCTGGCACACT | TCCACGGAGGACGGTGAGATG | 58 | 1 |  | c.6103-6449 | 347 |
| 25. | CAGTACGACGGGCTGGGTGAG | GCCACTGTTCTCCAGCAGGTA | 56 | 1.5 |  | c.6376-6783 | 408 |
| 26. | CTGCCGCTTCCTCTGCTATTT | CACCACCACATTGGCGTTCTC | 52 | 1.5 |  | c.6693-7059 | 367 |
| 27. | GCTGGAACCCCTGTGGTGGA | CCG GCACGAAGGATGCTGA | 56 | 1.5 |  | c.6965-7489 | 525 |
| 28. | TGGGCAAAGATGGGGCTCTG | TGGCGAACTCGTTGAGGATGG | 54 | 1.5 |  | c.7436-7912 | 477 |
| 29. | GCCTCATGTCGCTCTGCAGGTA | TTGTCGAAGGCCCACTTCTCG | 56 | 1.5 |  | c.7817-8309 | 493 |
| 30. | CACAGTGGATGCTGAAGGCAAC | GAGCGTGTCGTAGGGGACCA | 58 | 1.5 |  | c.8181-8733 | 553 |
| 31. | GAAGAAGAAGCAGGAGCTGGAAGC | TGGCGGACGAGAGCAGCAAG | 58 | 1.5 |  | c.8664-9163 | 492 |
| 32. | GCCACGCCTCTAACAAGGAG | GGCCGAAGCTTTTCCACATA | 49 | 1.5 |  | c.9086-9563 | 478 |
| 33. | CGACGTCCAGGTCTCTTGCTACCGAAC | GCCCGCGCTCCCACCATC | 62 | 1.5 |  | c.9477-9865 | 389 |
| 34. | AGTCAGGTGCCCGCTACA | GCAGAGCACAGAGAACTCGTC | 50 | 1.5 |  | c.9773-10206 | 434 |
| 35. | AGGAGGGCGAGCTGCTGGTG | CCCCGGCGCTTCTTCTTGGT | 59 | 1.5 |  | c.10163-10499 | 337 |
| 36. | AGATCAACAACATGTCCTTCCTG | ACTCTGCGCACGATTTTCTC | 50 | 1.5 |  | c.10388-10787 | 400 |
| 37. | TCTGCACAACAACCTTCACC | CAGTTTGCTCTTTTCCGTCAG | 50 | 1.5 |  | c.10656-11148 | 493 |
| 38. | CAGTTTTGAGGACCGCATGATAGA | CCCAGCTTCAGGGTGGAGGA | 56 | 1.5 |  | c.11001-11399 | 399 |
| 39. | GCAAGCACGGCTGCACAC | AGTCGCTGATGGATTCCTG |  | 1.5 |  | c.11295-11794 | 500 |
| 40. | GACACAGACAGGGAACACGACCAC | TCCTTGAGTTTCAGGAACATGTCGAA | 57 | 1.5 |  | c.11709-12206 | 498 |
| 41. | GCTCGTGGAATCCTCATCCAATGT | TCCTTCACCTGGGGCATCTCC | 57 | 1.5 |  | c.12138-12632 | 495 |
| 42. | CCTACCTGGGCCGCATCGAG | CCCTCCTCCGCGCCTTCC | 59 | 1.5 |  | c.12524-12833 | 310 |
| 43. | CCGAGGGCGAGCCGGAGA | CAGGCCGCCGCCGAACAG | 62 | 1.5 | GC-Rich reagent * | c.12758-13101 | 344 |
| 44. | AGTGGCGGCGCTGCTCTGG | TGGTTCCGGCTCGGGCTCTG | 68 | 1.5 | GC-Rich reagent * | c.12993-13491 | 499 |
| 45. | CCCGAGGGCTCTCCCATCCT | CTTCCATGTCGTCCTCCCCTGGT | 59 | 1.5 |  | c.13402-13783 | 382 |
| 46. | CCTGCGGTTCCTTGCCCTCT | CGGCGTGTTGAGCACCAGTC | 58 | 1.5 |  | c.13686-14130 | 445 |
| 47. | CACGGAGCAGCCTGAGGACGA | GGTAGACGACCACCGCCAGAAGG | 60 | 1.5 |  | c.14067-14551 | 485 |
| 48. | GCTGCGCACCATCCTGTCCT | TCCTTCACTTGCTCTTGTTGGTCTCG | 58 | 1.5 |  | c.14469-14858 | 390 |
| 49. | TCTGATCATCGACGCTTTTGGTGA | CACCTCTCCCCCAGCTGCCTTA | 58 | 1.5 |  | c.14805-15220 | 416 |

* GC-RICH PCR System Kit, Roche
